# Induction of *Bex* genes by curcumin is associated with apoptosis and activation of p53 in N2a neuroblastoma cells

**DOI:** 10.1038/srep41420

**Published:** 2017-02-01

**Authors:** Himakshi Sidhar, Ranjit K. Giri

**Affiliations:** 1 National Brain Research Centre, Manesar, Haryana 122051, India

## Abstract

Brain expressed X-linked (*Bex*) genes are newer group of pro-apoptotic genes. Role of any *Bex* gene in neuroblastoma and *Bex4* and *Bex6* in any cancer is completely unknown. Re-expression of all endogenous *Bex* genes by any nutraceutical is also unknown. Therefore, we investigated the induction of all endogenous *Bex* genes and associated mechanisms by curcumin using N2a, an aggressive neuroblastoma cell line. Curcumin induced all endogenous *Bex* genes prior to apoptosis in N2a cells in a dose- and time-dependent manner. Wortmannin (PI-3Kinases inhibitor), SP600125 (JNK inhibitor) and pifithrin-α (p53 inhibitor) abrogated curcumin-mediated induction of *Bex* genes. Inhibition of curcumin-mediated induction of *Bex* genes by pifithrin-α also inhibited N2a cells apoptosis suggesting, a direct role of *Bex* genes in N2a cells apoptosis and involvement of p53 in *Bex* genes induction. Curcumin treatment activated p53 through hyperphosphorylation at serine 15 before *Bex* genes induction indicating *Bex* genes are novel downstream targets of p53. Collectively, curcumin, a safe nutraceutical has the potential to induce all endogenous *Bex* genes to harness their anti-cancer properties in neuroblastoma cells. Re-expression of *Bex* genes by curcumin acts as tumor suppressors and may provide alternate strategy to treat neuroblastomas and other cancers with silenced *Bex* genes.

Neuroblastoma is the most common childhood cancer developed from uncommitted neural crest cells along the sympathetic nervous system and occasionally in central nervous system, including brain^1^,^2^. According to International Neuroblastoma Staging System, neuroblastomas are categorized into four different stages (I–IV)^3^. Stage-I and −II tumors either regress spontaneously or with minimal therapy and surgery, whereas the patients with stage-III or -IV tumors have poor prognosis as the cancer metastasizes to distant sites like lung, liver and bone marrow putting patients at higher risk for death^4^. Bansal *et al*., have reported that only 2 out of 101 neuroblastoma patients with stage-III or -IV remained disease free and in others, disease relapsed soon after completing OPEC therapy indicating the neuroblastomas were not sensitive to these chemotherapeutic agents^5^. Therefore, newer treatment methods, including the manipulation of endogenous genes/genome are warranted.

Epigenetic transcriptional repression has been reported in various cancers and takes place in tumor suppressor genes, pro-apoptotic genes and cell cycle regulators. Re-expression of these genes leads to suppression of tumor growth^6^,^7^. The *Bex* (Brain expressed X-linked) genes belong to a small family of genes including *Bex1/Rex3, Bex2/NADE5, Bex3/HGR74/NADE, Bex4* and *Bex6* in mouse while *Bex5* instead of *Bex6* in humans. All these genes are located on X-chromosome except *Bex6*, which is located on chromosome 16 of mouse genome^8^. These genes show high sequence similarities and are expressed majorly in brain^9^. All Bex proteins are highly dynamic in nature due to the presence of conserved disordered regions for which they participate in various cellular processes regulating neurodegeneration, cell cycle and tumor growth^10^,^11^. *Bex1* and *Bex2* have been identified as tumor suppressor genes and are silenced in malignant glioblastoma. Re-expression of *Bex1* or *Bex2* gene by transduction enhanced chemosensitization and apoptosis in glioblastoma cells^12^. *Bex3* has also been reported as a pro-apoptotic protein mediated by p75NTR^13^ and reduces tumor formation in mouse xenograft models of human breast cancer^14^. In addition, *Bex3*-mediated inhibition of DRG-1 induced PC12 cell proliferation suggests its tumor suppressor function^15^. Unlike these genes, report on pro-apoptotic role of *Bex4* gene is very limited and role of *Bex6* genes in any cancers has never been reported. Moreover, the role of any *Bex* genes is not studied in any neuroblastoma cells. It is highly impractical to re-express all *Bex* genes employing gene therapy in a variety of cancers and in various tissues simultaneously. Therefore, manipulating tumor cells genome by nutraceutical/s or pharmaceutical/s to re-express *Bex* genes can be of great importance in controlling cancer cells growth and death. Until today, there is no report on utilization of any small molecule or phytochemical to induce all the endogenous *Bex* genes.

Curcumin (diferuloylmethane), the principal curcuminoid of turmeric (*Curcuma longa*), has a wide range of applications in several biological processes including induction of apoptosis in various cancer cell types^16^,^17^. Curcumin has been known to kill N2a neuroblastoma cells through apoptosis^18^. However, use of curcumin to induce *Bex* genes is not reported. Therefore, we hypothesized that curcumin-mediated neuroblastoma cell death might induce *Bex* genes.

In the present study, induction of all endogenous *Bex* genes was explored using curcumin-mediated apoptosis in N2a neuroblastoma cells. Cell signaling inhibitors were employed to investigate the possible molecular mechanisms behind curcumin-mediated induction of *Bex* genes and to associate the expression of *Bex* genes with apoptotic neuroblastoma cells death. Collectively, our studies for the first time suggest that all the *Bex* genes can be induced specifically by curcumin to harness their tumor suppressor functions by inhibiting cell proliferation and activating apoptotic factors in N2a neuroblastoma cells.

## Results

### Curcumin induces apoptosis in N2a neuroblastoma cells in a dose-dependent manner

**Figure 1:**
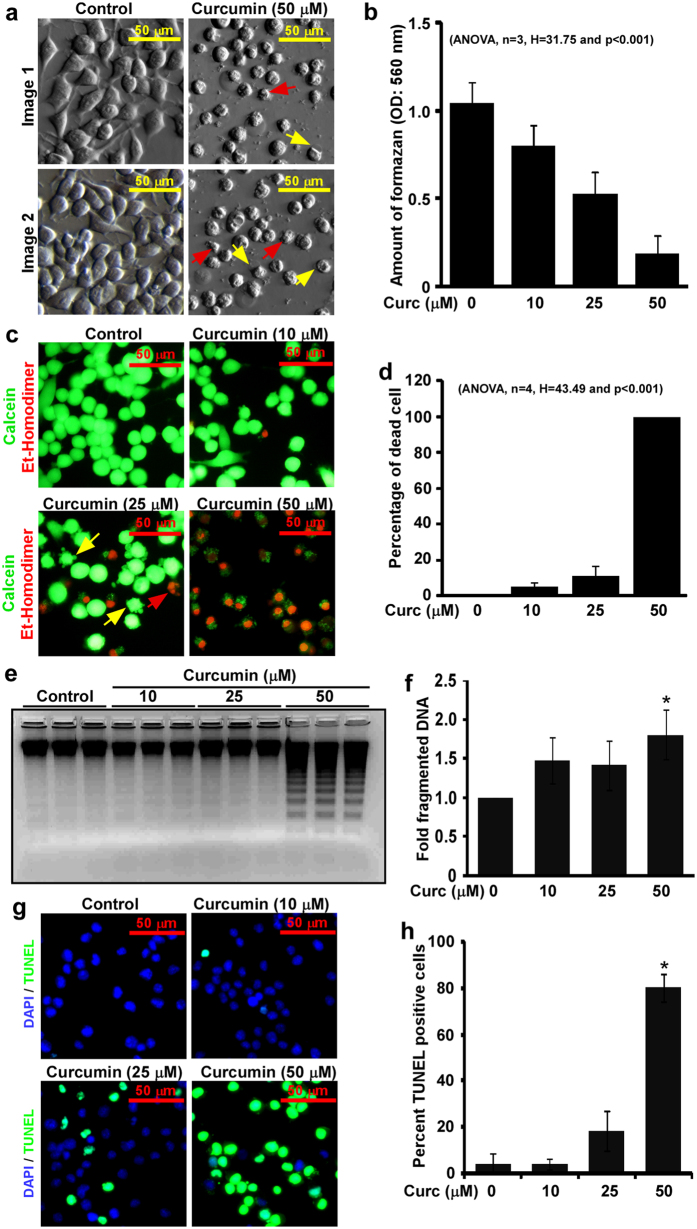
Curcumin induces apoptosis in N2a neuroblastoma cells. (**a**) Approximately, 80% N2a neuroblastoma cells were serum starved for 2 hours, treated with 50 μM of curcumin in serum free media for 24 hours and bright field imaging was done from three random fields. DMSO treated cells were used as controls. Images show increased cell deaths with membrane blebbing (yellow arrow) and nuclear condensation (red arrow) in curcumin treated N2a cells than controls. (**b**) MTT assays (see materials and methods) were performed on N2a cells treated with 10, 25, 50 μM of curcumin or with DMSO as controls for 24 hours. Amount of formazan produced was calculated and plotted as histograms, which indicate curcumin reduces N2a cell viability in a dose-dependent manner. (**c**) N2a cells used for LIVE/DEAD assay were cultured and treated as mentioned above. After 24 hours, fluorescent images were captured using 40X objective lens from three random fields at same settings. Images clearly exhibit decreased live cells (green) but increased dead cells (red) with membrane blebbing (yellow arrow) and chromatin fragmentation (red arrow) in a dose-dependent manner. (**d**) Percentage of dead cells from LIVE/DEAD imaging was calculated and plotted as histograms, which show curcumin kills N2a cells in a dose-dependent manner. (**e**) DNA fragmentation assay on N2a cells treated with 10, 25, 50 μM of curcumin or DMSO alone for 24 hours. Equal amount of DNA from all samples was resolved using agarose gel electrophoresis and imaged. The original gel image is shown in Supplementary Fig. S6. (**f**) Intensity of fragmented DNA to total DNA was measured and values were plotted as histograms showing increased oligonucleosomal DNA fragmentations in curcumin treated cells than controls. (**g**) N2a cells in culture were treated with curcumin similar to live-dead assay. After 24 hours, cells were collected and TUNEL assay was performed in suspension. Fluorescent images were captured from random fields at 40X magnification at same settings. (**h**) TUNEL positive cells was calculated in percentage and displayed as histograms. All histograms are displayed as mean ± standard deviation of three independent experiments and p-value displayed was calculated either using non-parametric one-way ANOVA or Student’s t-test. * = p ≤ 0.05 is considered statistically significant.

Bright field images exhibit curcumin-mediated N2a cells death. These images show membrane blebbing (yellow arrow) and nuclear condensation (red arrow) only in curcumin treated cells, which are commonly seen in apoptotic cell death (Fig. 1a). Results from MTT assay show, curcumin inhibited cell proliferation significantly in a dose-dependent manner, p < 0.001, n = 3 and H = 31.75 (Fig. 1b). Fluorescent images from LIVE/DEAD assays show a dose-dependent increased dead cells population in curcumin treated cells (Fig. 1c). These images also show increased membrane blebbing and fragmented nuclei in curcumin treated cells than control cells. Cell scoring analysis indicated 5 ± 2.3%, 11.4 ± 5% and 100% cell deaths in N2a cells treated with 10, 25 and 50 μM of curcumin respectively and the difference between the treatments is highly significant, p < 0.001, n = 4, H = 43.49 (Fig. 1d). DNA fragmentation assay demonstrated increased DNA fragmentation in curcumin treated N2a cells than controls with maximum at 50 μM (Fig. 1e). Densitometric analysis demonstrated approximately 1.8 ± 0.31-fold increased DNA fragmentation (p < 0.05) in cells treated with 50 μM of curcumin than control cells (Fig. 1f) suggesting curcumin induces large-scale genetic lesions during N2a cells death. TUNEL assay, a widely used method to detect apoptosis was also performed. Fluorescent images from TUNEL assay show dose-dependent increased TUNEL positive cells in curcumin treated N2a cells than controls (Fig. 1g). Cell count analysis indicated approximately 3.9 ± 4.2, 3.9 ± 2.3, 18.06 ± 8.56 and 80 ± 6.07% TUNEL positive cells in control, 10, 25 and 50 μM of curcumin treated N2a cells respectively (Fig. 1h). These data suggest that curcumin-mediated N2a cells death involves apoptosis. To avoid 100% cell death in 50 μM of curcumin treatment, 25 μM of curcumin treatment was used in subsequent experiments.

### Curcumin inhibits ERK1/2 activation but activates intrinsic apoptotic pathway in N2a cells

**Figure 2:**
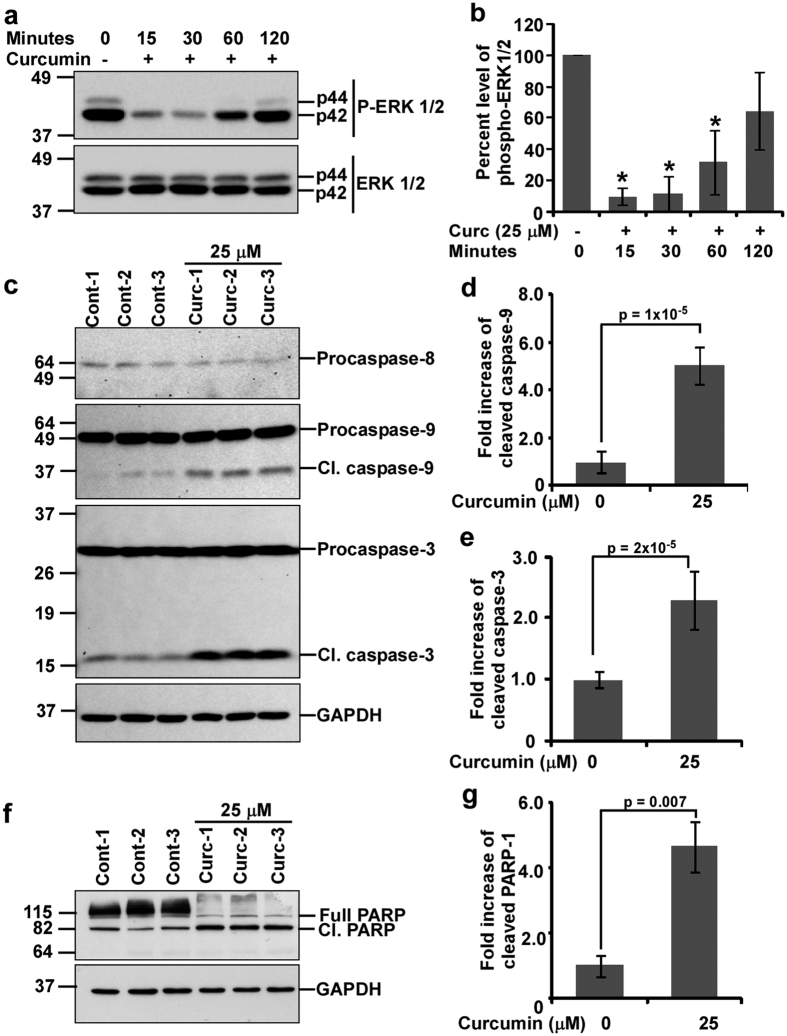
Curcumin inhibits ERK1/2 phosphorylation and induces cell death through intrinsic apoptotic pathway in N2a cells. (**a**) Approximately 70–80% confluent N2a cells cultured in 25 cm^2^ flasks were serum starved for 2 hours and treated with 25 μM of curcumin in serum free media. DMSO treated cells were used as controls. Total cell lysates were obtained after indicated time points and western blot analysis was performed using anti-phospho-ERK1/2 antibody. Blots were stripped and re-immunoblotted with anti-ERK1/2 antibody. (**b**) Intensities of phosphorylated ERK1/2 bands were normalized with corresponding ERK1/2 band intensities and histograms were plotted as mean ± standard deviation of three independent experiments, which show curcumin significantly inhibited ERK1/2 phosphorylation with maximum at 30 minutes. (**c**) Approximately 60–70% confluent N2a cells were serum starved for 2 hours and treated with 25 μM of curcumin for 24 hours. Equivalent amount of DMSO was treated in control cells. Western blot analysis was performed utilizing anti-caspase-8, 9 or 3 antibodies indicating increased proteolysis of caspases-9 and -3 but not caspase-8 in curcumin treated N2a cells than controls at 24 hours. Blots were stripped and immunoblotted with anti-GAPDH antibody. Densitometric analysis of cleaved caspase-9 (**d**) and caspase-3 (**e**) are plotted as histograms. (**f**) Western blot analysis was performed on control and 25 μM of curcumin treated N2a cells at 24 hours of treatment utilizing anti-PARP-1 antibody which indicates increased proteolysis of PARP-1 in curcumin treated N2a cells. (**g**) Level of cleaved PARP-1 (89 kDa) was calculated after normalizing its intensity to total PARP-1 and GAPDH. Values obtained are plotted as histograms of mean ± standard deviation from three independent experiments. P-values displayed were calculated using two tailed unpaired, Student’s t-test. * = p ≤ 0.05 is considered statistically significant. The original immunoblot images are shown in Supplementary Fig. S7 (for **a**) and Supplementary Fig. S8 (for **c**,**f**).

Activation of extracellular signal-regulated kinases (ERK1/2) through hyperphosphorylation is a key feature in almost all cancers^19^. Hence, inactivation of ERK1/2 along with activation of apoptotic factors is necessary in curcumin-mediated neuroblastoma cell death. Therefore, the phosphorylation status of ERK1/2 was studied and western blot analysis of ERK1/2 phosphorylation exhibited decreased ERK1/2 phosphorylation at all the time points studied with maximum at 30 minutes of curcumin treatment (Fig. 2a). Densitometric analysis of phosphorylated ERK1/2 normalized with ERK1/2 demonstrated approximately 90.1 ± 5.1%, 88.4 ± 11.1%, 68.3 ± 20.3% and 35.5 ± 24.8% decrease in ERK1/2 phosphorylation at 15, 30, 60 and 120 minutes of curcumin treatment respectively than controls (Fig. 2b), suggesting curcumin rapidly inhibits mitotic cell signals and prepares the cells towards apoptotic cell death. In order to confirm apoptotic cell deaths in curcumin treated N2a cells, activation of caspases were studied. Proteolytic activation of caspases are key features of apoptosis^20^. Western blot analysis indicated increased proteolytic activation of caspase-9 but not caspase-8 in curcumin treated cells than corresponding controls (Fig. 2c). Densitometric analysis of cleaved caspase-9 indicated 5.04 ± 0.79-fold increased cleaved products in curcumin treated cells than controls (Fig. 2d). Furthermore, caspase-3 is also activated in curcumin treated cells (Fig. 2c). Densitometric analysis indicated significant increased cleaved caspase-3 level (2.29 ± 0.47-fold, p = 0.00002) in curcumin treated cells than controls (Fig. 2e). Western blotting (Fig. 2f) and densitometric analysis (Fig. 2g) of poly (ADP Ribose) polymerase (PARP-1) fragmentation showed significant increased PARP-1 proteolysis (4.65 ± 0.77-fold, p = 0.007) in curcumin treated cells than controls. Collectively, these data suggest curcumin-mediated N2a cells death involves intrinsic but not extrinsic apoptotic pathway.

### Curcumin induces *Bex* genes in N2a neuroblastoma cells in a dose-dependent manner

**Figure 3:**
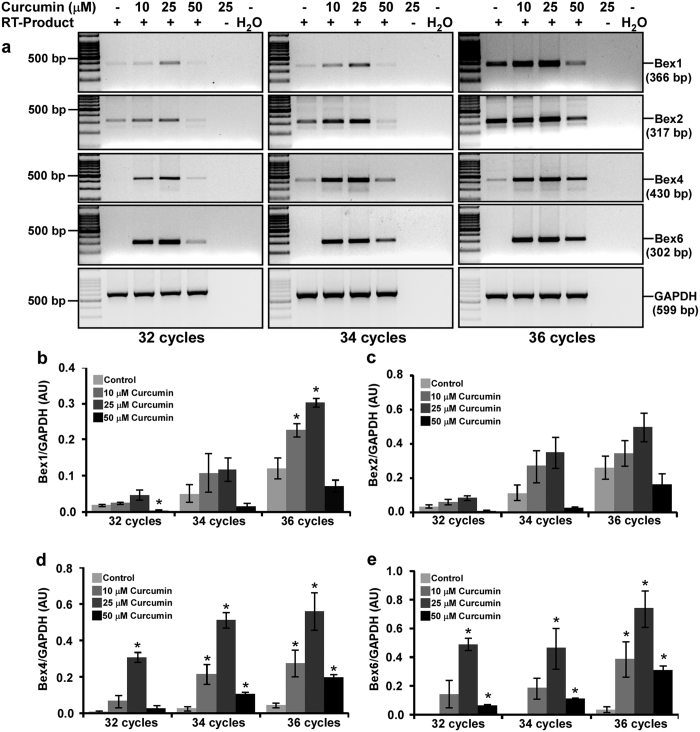
Curcumin induces *Bex* genes in N2a neuroblastoma cells in a dose-dependent manner. (**a**) N2a cells (3 × 10^5^ cells) were cultured for two days in 25 cm^2^ flask, serum starved for 2 hours and treated either with 10, 25 or 50 μM of curcumin or with equal amount of DMSO (controls) in serum free media for 2 hours. Total RNA was isolated by Trizol reagent and treated with DNase I to remove any DNA contamination. Reverse transcription on 5 μg of DNA-free RNA was performed and Bex cDNAs were amplified either for 32, 34 or 36 PCR cycles. Agarose gel electrophoresis shows a dose-dependent induction of *Bex* genes by curcumin. The original gel images are shown in Supplementary Fig. S9. Densitometric analysis of Bex1 (**b**), Bex2 (**c**), Bex4 (**d**) and Bex6 (**e**) mRNA amplicons was performed using Image lab software and values obtained were normalized with respective GAPDH band intensity, and were plotted as histograms of mean ± standard error of mean from three independent experiments. P-values displayed were calculated by two-tailed, unpaired Student’s t-test and * = p ≤ 0.05 is considered statistically significant.

Reverse transcribed-polymerase chain reaction (RT-PCR) analysis of *Bex* genes expression demonstrated *Bex1, Bex2, Bex4* and *Bex6* genes were induced differentially to varying amounts of curcumin with maximum at 25 μM at 2 hours of treatment (Fig. 3a), whereas GAPDH expression remain unchanged across samples and PCR cycles. Densitometric analysis demonstrated 25 μM of curcumin treatment upregulated *Bex1* expression approximately by 2.3, 2.2 and 2.4-fold and *Bex2* expression approximately by 2.3, 2.9, and 1.8-fold at 32, 34 and 36 cycles respectively than control cells (Fig. 3b and c respectively). Moreover, *Bex4* expression was upregulated by 42, 22, and 12-fold at 32, 34 and 36 cycles respectively and *Bex6* expression was upregulated by 212, 418, and 22-fold at 32, 34 and 36 cycles respectively in curcumin treated cells than control cells (Fig. 3d and e respectively). Intensity of Bex PCR products increased with increase in PCR cycle numbers suggesting the intensities are below saturation at 32 and 34 cycles but approaching saturation by 36 cycles, which justify quantification of *Bex* genes expression.

### Curcumin induces *Bex* genes in N2a neuroblastoma cells in a time-dependent manner

**Figure 4:**
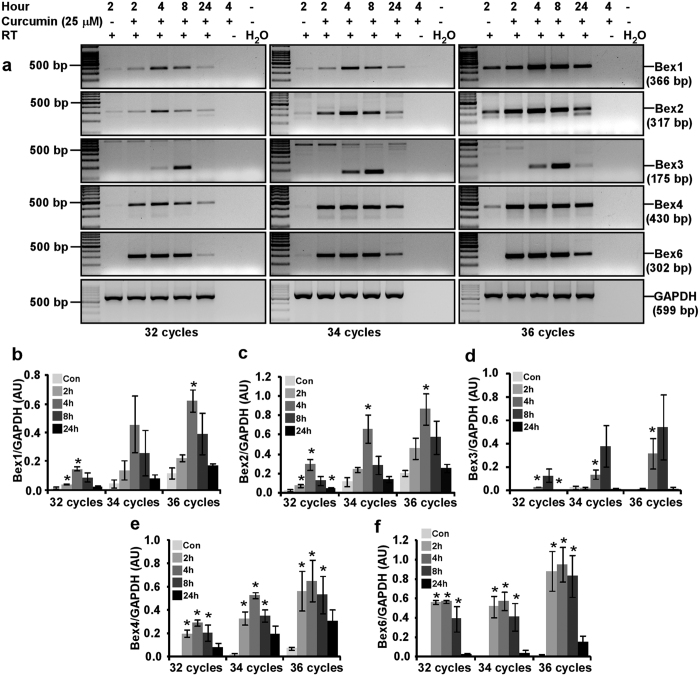
Optimization of curcumin treatment duration for the induction of *Bex* genes in N2a cells. (**a**) N2a cells (3 × 10^5^ cells) were cultured for two days in 25 cm^2^ flask, serum starved for 2 hours and treated with 25 μM of curcumin for 2, 4, 8 or 24 hours. Control cells were treated with equivalent amount of DMSO for 2 hours. Total RNA was isolated at indicated time points using Trizol reagent and treated with DNase I for 15 minutes at room temperature. RT-PCR analysis of DNA-free Bex mRNA was performed for 32, 34 or 36 PCR cycles. Agarose gel electrophoresis clearly demonstrates a time-dependent induction of *Bex* genes by curcumin. The original gel images are shown in Supplementary Fig. S10. Intensity of Bex1 (**b**), Bex2 (**c**), Bex3 (**d**), Bex4 (**e**) and Bex6 (**f**) PCR products were obtained and normalized with corresponding GAPDH band intensity, and displayed as histograms of mean ± standard error of mean from three independent experiments. P-values displayed were calculated by using two-tailed, unpaired Student’s t-test and * = p ≤ 0.05 is considered statistically significant.

To establish optimal duration of curcumin stimulation on *Bex* genes, we performed time course experiments ranging from 2 to 24 hours using semi-quantitative RT-PCR analysis. Expression of *Bex1, Bex2, Bex4*, and *Bex6* genes was upregulated at all the time points with a maximal induction at 4 hours. Intensity of GAPDH band did not change markedly between treated and control samples (Fig. 4a). Densitometric analysis at 4 hours of curcumin treatment exhibits *Bex1* expression was upregulated by 8.8, 9.7 and 5.2-fold and *Bex2* was overexpressed by 10.4, 5.6 and 4.1-fold at 32, 34 and 36 cycles respectively than controls (Fig. 4b and c respectively). Similarly, *Bex4* was up regulated by 106.5, 22.6 and 8.9-fold, and *Bex6* was up regulated by 291.4, 135.9 and 71.5-fold at 32, 34 and 36 cycles respectively in curcumin treated cells than controls (Fig. 4e and f respectively). Curcumin also induced *Bex3* gene with maximum at 8 hours of treatment. Unlike other *Bex* genes, *Bex3* induction by curcumin was not observed at 2 hours of treatment (Fig. 4a). Densitometric analysis demonstrated that curcumin upregulated *Bex3* gene by 30.8, 14.2 and 196.7-fold at 32, 34 and 36 cycles respectively than control N2a cells (Fig. 4d). Taken together, curcumin has the potential to induce all the *Bex* genes over a long period.

### Curcumin specifically induces *Bex* genes in N2a neuroblastoma cells

**Figure 5:**
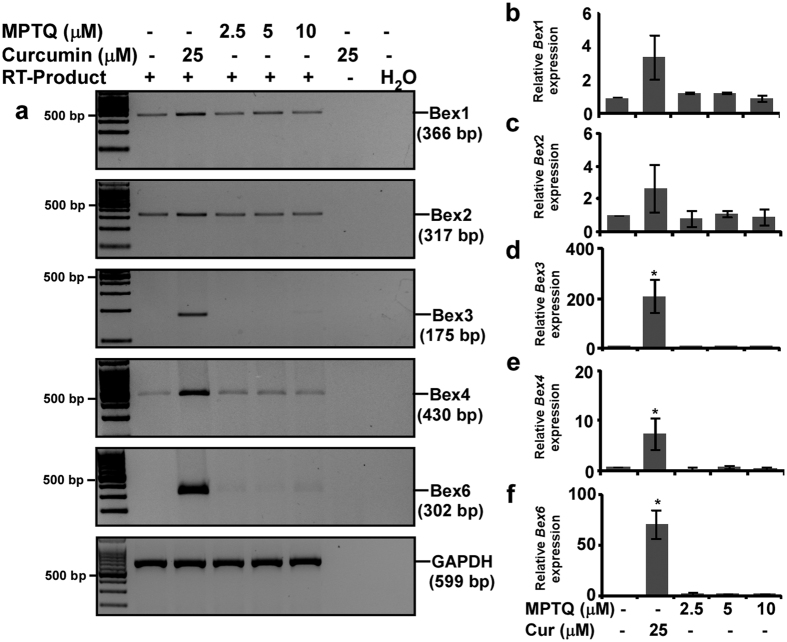
Curcumin is a specific inducer of all the *Bex* genes. (**a**) Approximately 80% confluent N2a cells were serum starved for 2 hours and treated with either 2.5, 5, 10 μM of MPTQ or 25 μM of curcumin in serum free DMEM for 4 hours. Equivalent amount of DMSO treated N2a cells were considered as controls. Total RNA was isolated, DNase I treated and expression of Bex mRNAs was studied by RT-PCR analysis. GAPDH was used as loading control. The original gel images are shown in Supplementary Fig. S11. Densitometric analysis of Bex1 (**b**), Bex2 (**c**), Bex3 (**d**), Bex4 (**e**) and Bex6 (**f**) PCR products was performed and normalized with corresponding GAPDH band intensity. Values are displayed as histograms of mean ± standard deviation from three independent experiments, which indicates induction of all endogenous *Bex* genes are observed in curcumin but not in MPTQ treated N2a cells. P-values displayed were calculated by using two-tailed, unpaired Student’s t-test and * = p ≤ 0.05 is considered statistically significant.

Whether induction of *Bex* genes is specific to curcumin-mediated N2a cell apoptosis or a general consequence of apoptosis by any anti-cancer agent, we utilized both curcumin and 8-Methoxypyrimido [4′,5′:4,5]thieno(2,3-b) Quinoline-4(3 H)-One (MPTQ) to induce *Bex* genes. MPTQ is a new anti-cancer agent. It induces apoptosis in different cancer cell lines^21^ and through intrinsic apoptotic pathway in mouse and human neuroblastoma cells^22^. Both 10 μM of MPTQ and 25 μM of curcumin induce N2a neuroblastoma cells apoptosis to a similar amount, 15%, and 11% respectively. Therefore, we utilized 10 μM of MPTQ in parallel with 25 μM of curcumin to understand whether induction of *Bex* genes is associated with any type of apoptosis or specific to curcumin. Unlike curcumin, MPTQ failed to induce any *Bex* genes at various doses tested in N2a cells (Fig. 5a). Densitometric analysis showed 3.03 ± 1.4, 2.68 ± 1.4, 209 ± 67.7, 7.56 ± 3.2 and 70.9 ± 3.4-fold increased expression of *Bex1, Bex2, Bex3, Bex4* and *Bex6* genes respectively in curcumin treated cells than controls, whereas expression of all these *Bex* genes in MPTQ treated N2a cells was comparable to control cells (Fig. 5b–f). Furthermore, higher dose of MPTQ (25 μM) also failed to induce any *Bex* genes as compared to curcumin (Supplementary Fig. S1). In addition, effect of tunicamycin (TM), a well-known inducer of endoplasmic reticulum stress, on *Bex* gene induction in N2a cells was also studied. Results demonstrate that toxic dose of TM (0.1 μg/ml) failed to induce *Bex* genes as efficiently as curcumin (as shown in Supplementary Fig. S2). The expression of *Bex3, 4* and *6* in TM treated N2a cells is comparable to controls. Therefore, induction of all *Bex* genes is a response, specific to curcumin in N2a neuroblastoma cells.

### Curcumin-mediated induction of *Bex* genes involves PI3-kinases, JNK and p53 in N2a neuroblastoma cells

**Figure 6:**
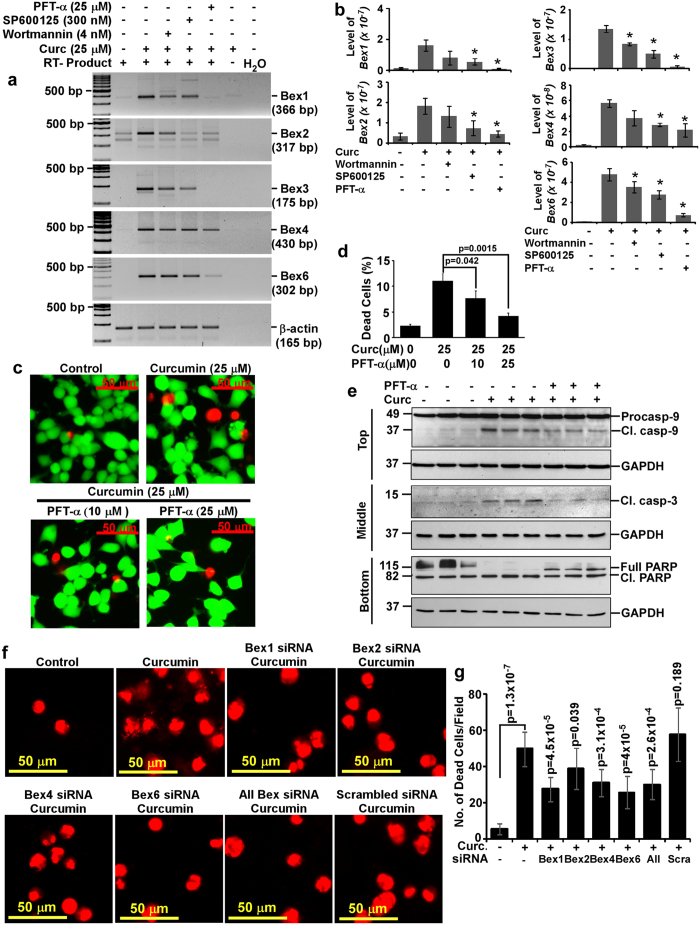
Molecular mechanisms involved in the induction of *Bex* genes and its association with curcumin-mediated N2a neuroblastoma cells apoptosis. (**a**) Approximately 80% confluent N2a cells were treated with wortmannin (4 nM), SP600125 (300 nM) or pifithrin-α (25 μM) for 30 minutes after two hours of serum starvation. Cells were then treated with either curcumin (25 μM) alone or along with these inhibitors. DMSO treated cells were used as controls. Total RNA was isolated at 4 hours of curcumin treatment. DNA-free RNA was used for RT-PCR analysis as described earlier, which demonstrates wortmannin, SP600125 and pifithrin-α abrogate curcumin-mediated induction of *Bex* genes. The original gel images are shown in Supplementary Fig. S12. (**b**) Intensities of Bex1, Bex2, Bex3, Bex4 and Bex6 PCR products were measured and normalized values were displayed as histograms of mean ± standard error from three independent experiments. (**c**) LIVE/DEAD analysis was performed as described earlier except pifithrin-α was treated 30 minutes before curcumin treatment. Multiple images were captured and displayed with equal image settings. (**d**) Percentage of dead cells in each group was calculated from three independent experiments and plotted as histograms. (**e**) Cell lysates were prepared after 24 hours either from DMSO or curcumin (25 μM) alone or along with pifithrin-α (25 μM) treated N2a cells. Western blotting was performed using antibodies against caspase-9, caspase-3 or PARP1. GAPDH immunoblotting was used as loading and transfer control. The original immunoblot images are shown in Supplementary Fig. S13. (**f**) N2a cells were transfected with Bex1, 2, 4 or 6 siRNA duplexes either individually or in combination for 72 hours followed by curcumin (25 μM) treatment for 24 hours. Scrambled siRNA transfected N2a cells were used as negative controls. Untransfected N2a cells treated with 25 μM of curcumin or DMSO were used as positive and negative controls respectively. LIVE/DEAD assay was performed at 24 hours of curcumin treatment and fluorescent images from nine random fields were captured at 30X magnification indicating inhibition of Bex siRNAs against curcumin-mediated cell death. (**g**) The number of dead cells (red) were counted manually and plotted as histograms. P-values displayed as numbers or * were calculated using two tailed unpaired Student’s t-test. P-value ≤ 0.05 is considered statistically significant.

Pharmacological inhibitors against MEK1/2 (PD98059 and U0126), p38 MAP Kinase (SB202190), PI3-Kinases (wortmannin), c-Jun N-terminal kinases (SP600125) and p53, the guardian of genome (pifithrin-α) were used to study the mechanisms behind curcumin-mediated induction of *Bex* genes. Among all these inhibitors, PD98059, U0126 and SB202190 at IC_50_ dose failed to abrogate curcumin-mediated induction of *Bex* genes indicating ERK1/2 and p38 MAP kinases are not involved in curcumin-mediated induction of *Bex* genes. However, SP600125 and wortmannin showed marginal inhibitory effect against curcumin-mediated induction of some *Bex* genes (Supplementary Fig. S3a,b), which warranted optimization of their dose. Therefore, N2a neuroblastoma cells were treated with varying amount of wortmannin (4, 8 or 12 nM), SP600125 (100, 200 or 300 nM) and pifithrin-α (5, 10, 15, 20 or 25 μM). The results show, inhibitory effect of SP600125, wortmannin and pifithrin-α was maximum at 300 nM, 4 nM and 25 μM respectively (Supplementary Fig. S3c–g) and were used in subsequent experiments employing these inhibitors. Semi-quantitative RT-PCR analysis demonstrated curcumin-mediated induction of *Bex1* (~10-fold), *Bex2* (~5.5-fold), *Bex3* (~336-fold), *Bex4* (~25-fold) and *Bex6* (~138-fold) genes was inhibited by 4 nM of wortmannin, 300 nM of SP600125 and 25 μM of pifithrin-α (Fig. 6a). Intensity of Bex PCR products were measured and normalized with corresponding β-actin along with 28 S and 18 S bands of DNase I treated RNA. Analysis showed that wortmannin reduced *Bex1, 2, 3, 4* and *6* genes expression by 64.97 ± 52.4%, 48.25 ± 32.4%, 37.7 ± 11.6%, 37.05 ± 35.4% and 25.3 ± 19.6% respectively as compared to curcumin treated N2a cells. SP600125 reduced *Bex1, 2, 3, 4* and *6* genes expression by 75.2 ± 15.1%, 88.5 ± 38%, 64.4 ± 12.1%, 50.8 ± 8.4% and 40.4 ± 15.4% respectively to curcumin treated N2a cells. Moreover, pifithrin-α strongly reduced *Bex1, 2, 3, 4* and *6* genes expression by 103.3 ± 0.4%, 90.7 ± 14.4%, 96.4 ± 5.6%, 62.8 ± 33.3% and 85 ± 6.5% respectively as compared to curcumin treated N2a cells (Fig. 6b). Thus, inhibition of curcumin-mediated *Bex* gene induction by wortmannin, SP600125 and pifithrin-α strongly suggest the involvement of PI3-kinase, c-Jun N-terminal kinases (JNKs) and p53-associated pathways.

### Association of *Bex* genes induction with curcumin-mediated N2a neuroblastoma cells apoptosis

Involvement of *Bex* genes in cancer cell apoptosis warrants experiments that reduce *Bex* genes expression and correlates it with cancer cells death. Since curcumin-mediated induction of *Bex* genes was inhibited strongly by pifithrin-α, therefore, we wanted to study whether pifithrin-α-mediated inactivation of *Bex* genes also abrogates apoptosis in curcumin treated N2a cells. LIVE/DEAD assays demonstrated curcumin-mediated N2a cell deaths were reversed by pifithrin-α in a dose-dependent manner (Fig. 6c). Cell scoring analysis showed approximately 11 ± 1.4% cells death in curcumin treated N2a cells over control cells (2.3 ± 0.3%) at 24 hours, which was reduced significantly in a dose-dependent manner to 7.7 ± 1.4% and 4.2 ± 0.6% in 10 and 25 μM of pifithrin-α pretreated cells respectively (Fig. 6d). In addition, pifithrin-α also inhibited curcumin-mediated activation of apoptotic factors, such as caspase-9, caspase-3 and proteolysis of PARP-1 (Fig. 6e top, middle and bottom panel respectively). In addition, N2a cells transfected with siRNA duplexes against Bex transcripts either individually or in combination showed inhibition towards curcumin-mediated N2a cell deaths (Fig. 6f). Analysis of ethidium homodimer positive (indicator of dead cell population) cells suggests siRNA against Bex6 and Bex1 transcripts have maximum inhibition followed by cocktail of all siRNAs, Bex4 and Bex2 in curcumin mediated N2a cell deaths (Fig. 6g). In order to authenticate our imaging strategy, images were also obtained from 4X objective lens to cover more area and from the center of the well where dead cells have a tendency to accumulate. Intensity from these images were obtained and plotted as histograms. Our results again suggest siRNA against Bex1 and Bex6 transcripts have maximum inhibitory effect followed by cocktail of all siRNAs and Bex4 in curcumin-mediated N2a cell deaths (Supplementary Fig. S4). However, scrambled siRNA transfected N2a cells showed cell deaths comparable to curcumin treated N2a cells in both the experimental settings discussed. Collectively, all these results provide a strong evidence for the functional role of *Bex* genes in N2a neuroblastoma cell apoptosis and possible involvement of p53 in curcumin-mediated induction of *Bex* genes.

### Activation of p53 through hyperphosphorylation at ser15 in curcumin treated N2a cells prior to *Bex* genes induction

**Figure 7:**
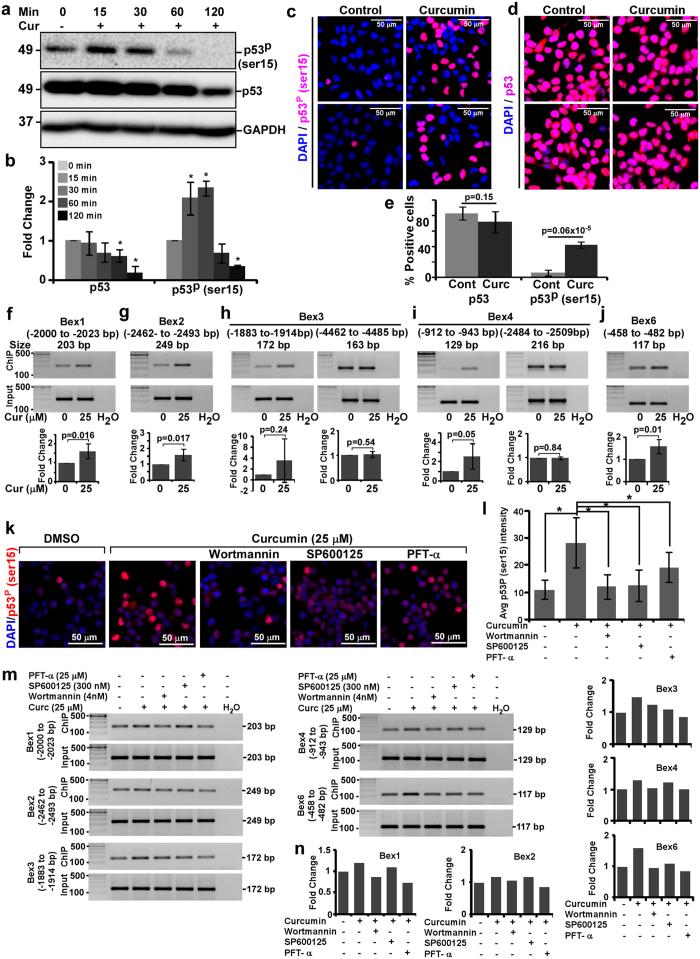
Hyperphosphorylation of p53 at ser15 and its association with curcumin-mediated *Bex* gene induction in N2a cells. (**a**) Western blot analysis of p53 phosphorylation at ser15 in curcumin (25 μM)-treated N2a cells at indicated time points using anti-phospho-p53-ser15, anti-p53 or anti-GAPDH antibodies. The original immunoblot images are shown in Supplementary Fig. S14. (**b**) Fold change of p53 and phospho-p53-ser15 level after normalizing with GAPDH level was calculated and plotted as histograms of mean ± standard deviation from three independent experiments. Immunofluorocytochemical analysis of phospho-p53-ser15 (**c**) and p53 (**d**) level at 30 minutes of curcumin treated or untreated N2a cells. (**e**) Percent phospho-p53-ser15 and p53 positive cells were calculated in both the groups and plotted as histograms. (**f**) Interaction of activated p53 to the promoters of *Bex* genes by chromatin immunoprecipitation (ChIP) assay. N2a cells were treated with curcumin (25 μM) for 30 minutes followed by ChIP using anti-phospho-p53-ser15 antibody. PCR amplification and densitometric analysis (see material and methods) of p53 binding elements of *Bex1* (**f**), *Bex2* (**g**), *Bex3* (**h**), *Bex4* (**i**) and *Bex6* (**j**) genes to phospho-p53-ser15 show at least one p53 binding element of each *Bex* gene interact more with phospho-p53-ser15 in curcumin treated N2a cells than corresponding controls. (**k**) Immunofluorocytochemical analysis demonstrates wortmannin, SP600125 and pifithrin-α reduces p53-ser15 phosphorylation at 30 minutes of curcumin treated N2a cells. (**l**) Average nuclear phospho-p53-ser15 intensities with or without inhibitors were measured and plotted as histograms from three independent experiments. The original gels images used for f-j are shown in Supplementary Fig. S15. (**m**) Effect of wortmannin (4 nM), SP600125 (300 nM) and pifithrin-α (25 μM) on phospho-p53-ser15 DNA binding activity to *Bex* genes at 30 minutes of curcumin (25 μM) treated N2a cells. Chromatin immunoprecipitation was performed as described above. PCR amplification was performed corresponding to p53 binding element/s of *Bex1, Bex2, Bex3, Bex4* and *Bex6* gene promoters. Input DNA PCR was also used as loading control. The original gel images are shown in Supplementary Fig. S16. (**n**) Band intensities of ChIP PCR products were normalized with corresponding input DNA PCR products and fold change was calculated. Values were plotted as histograms. P-values displayed were calculated using two-tailed, unpaired Student’s t-test and * = p ≤ 0.05 is considered statistically significant.

Inhibition of curcumin-mediated induction of *Bex* genes by pifithrin-α, an established inhibitor of p53^23^ and increased TUNEL positive cells in curcumin treated N2a cells hint the involvement of p53. Therefore, activation of p53 by hyperphosphorylation at ser15 was investigated. Western blot analysis demonstrates increased p53-ser15 phosphorylation after 15 and 30 minutes of curcumin treatment, which decreased thereafter (Fig. 7a, top panel). Unlike increased p53-ser15 phosphorylation, the level of p53 decreased in a time-dependent manner in curcumin treated N2a cells than controls (Fig. 7a, middle panel). Densitometric analysis of phospho-p53-ser15 intensity demonstrated significant increased p53 phosphorylation by 2.08 ± 0.4 and 2.33 ± 0.18-fold after 15 and 30 minutes of curcumin treatment respectively. By 2 hours, disappearance of phospho-p53-ser15 level reached statistically significant (Fig. 7b, right panel). Densitometric analysis also indicated that p53 level decreased significantly by 37.8 ± 15.3% and 80.6 ± 15.4% after 1 and 2 hours respectively but marginally after 15 and 30 minutes of curcumin treatment (Fig. 7b, left panel). Images from immunocytochemistry exhibit increased nuclear phospho-p53-ser15 intensity in curcumin treated cells than control cells (Fig. 7c). Cell count analysis show approximately 42 ± 4.25% and 6.4 ± 3.8% phospho-p53-ser15 positive cells in curcumin treated and control N2a cells respectively and the difference between the treatments is highly significant, p = 6E-07 (Fig. 7e). However, the number of p53 positive cells shows a marginal decrease in curcumin treated N2a cells than control cells at 30 minutes of treatment (Fig. 7d and e), which paralleled the results from western blot analysis at similar time point.

### Chromatin immunoprecipitation demonstrates p53-mediated regulation of *Bex* genes in curcumin treated N2a neuroblastoma cells

Curcumin-mediated increase in phosphorylation of p53 at ser15 prior to *Bex* gene induction urged us to study its binding on *Be*x gene promoters after 30 minutes of treatment. The putative binding sites of p53 were identified manually in promoter (6000 bp upstream of transcription start site) region of *Bex1, 2, 3, 4* and *6* (Supplementary Fig. S5). Results from ChIP assay using anti-phospho-53-ser15 antibody demonstrated an increased amplification of PCR products corresponding to p53 binding sites on *Bex1* (−2000 to −2023 bp), *Bex2* (−2462 to −2493 bp), *Bex3* (−1883 to −1914 bp), *Bex4* (−912 to −943 bp) and *Bex6* (−458 to −482 bp) promoters in curcumin treated samples as compared to controls. However, binding of phospho-p53-ser15 at other sites on *Bex3* (−4462 to −4485 bp) and *Bex4* (−2484 to −2509 bp and −1955 to −1977) promoters was not affected post curcumin treatment. Input DNA showed comparable amplification in control and curcumin treated samples (Fig. 7f–j respectively). Densitometric analysis of PCR amplicons was performed and intensities of ChIP DNA amplicons was normalized with input DNA amplicons. The fold change between two groups was calculated and histograms were plotted indicating 1.61 ± 0.37 (p = 0.01), 1.59 ± 0.36 (p = 0.01), 3.57 ± 3.94 (p = 0.24), 2.55 ± 1.33 (p = 0.05) and 1.57 ± 0.31 (p = 0.01) fold more phospho-p53-ser15 binding to *Bex1, Bex2, Bex3, Bex4* and *Bex6* promoters respectively in curcumin treated cells than controls (Fig. 7f–j respectively). Thus, our results clearly indicate, induction of all endogenous *Bex* genes in curcumin treated N2a neuroblastoma cells are regulated by p53.

### Wortmannin, SP600125 and pifithrin-α inhibited p53-ser15 phosphorylation and it’s binding to *Bex* genes promoter in curcumin treated N2a neuroblastoma cells

Since, pretreatment of N2a cells with wortmannin (4 nM), SP600125 (300 nM) or pifithrin-α (25 μM) inhibited curcumin-mediated induction of *Bex* genes, we therefore investigated their effect on p53-ser15 phosphorylation prior to the induction of *Bex* genes. Immunofluorescent images demonstrate curcumin-mediated p53-ser15 phosphorylation is reduced notably in wortmannin and SP600125 but to lesser level in pifithrin-α pretreated N2a cells than cells treated with curcumin only (Fig. 7k). Average nuclear phospho-p53-ser15 intensity shows approximately 2.57-fold increased p53-ser15 phosphorylation in curcumin treated cells than controls and the difference between untreated and curcumin treated samples is highly significant (p = 4E−08). Wortmannin, SP600125 and pifithrin-α inhibited curcumin-mediated p53-ser15 phosphorylation significantly by 92.2%, 91.4% and 52.6% respectively than N2a cells treated with curcumin only (Fig 7l). Furthermore, we investigated the effect of these inhibitors on curcumin-mediated binding of phospho-p53-ser15 onto *Bex* gene promoters prior to the induction of *Bex* genes. Results from chromatin immunoprecipitation showed that curcumin-mediated binding of phospho-p53-ser15 to *Bex1* (−2000 to −2023 bp), *Bex2* (−2462 to −2493 bp), *Bex3* (−1883 to −1914 bp), *Bex4* (−912 to −943 bp) and *Bex6* (−458 to −482 bp) promoters is reduced in wortmannin and pifithrin-α but not much in SP600125 pretreated N2a cells than cells treated with curcumin only (Fig. 7m). However, binding of phospho-p53-ser15 to *Bex3* (−4462 to −4485 bp), and *Bex4* (−1956 to −1977 and −2484 to −2509 bp) promoters was similar in inhibitor pretreated and only curcumin treated N2a cells (data not shown). Analysis of intensity of phospho-p53-ser15 ChIP DNA PCR products normalized with corresponding input DNA PCR products intensity demonstrated wortmannin reduced phospho-p53-ser15 chromatin binding by 166.60, 53.06, 49.17, 84.81 and 108.70% to *Bex1* (−2000 to −2023 bp), *Bex2* (−2462 to −2493 bp), *Bex3* (−1883 to −1914 bp), *Bex4* (−912 to −943 bp) and *Bex6* (−458 to −482 bp) gene promoters respectively as compared to curcumin treated N2a cells. SP600125 reduced binding of phospho-p53-ser15 by 58.4, 3.79, 81.7, 22.04 and 82.44% to the same region of *Bex1, Bex2, Bex3, Bex4* and *Bex6* gene promoters respectively as compared to curcumin treated N2a cells. Pifithrin-α inhibited phospho-p53-ser15 chromatin binding by 228, 197.15, 133.20, 97.91 and 126.0% to the said region of *Bex1, Bex2, Bex3, Bex4* and *Bex6* gene promoters respectively as compared to curcumin treated N2a cells (Fig. 7n). Collectively, these results clearly indicate the direct binding of phospho-p53-ser15 onto *Bex* genes promoters in curcumin treated N2a neuroblastoma cells.

## Discussion

Re-expression/induction of all endogenous *Bex* genes by a safe and small molecule was our main interest. In addition, we also wanted to investigate their function in neuroblastoma, which is completely unknown. Our results clearly demonstrated that curcumin, the active principle of turmeric has the potential to induce *Bex1* and *Bex2* and re-express *Bex3, Bex4* and *Bex6* genes. Induction of these genes functions as tumor suppressors in N2a neuroblastoma cells.

At first, we established a robust curcumin-mediated apoptosis in N2a neuroblastoma cells. In this study, we observed the cytotoxic effect of curcumin on N2a cells similar to earlier studies^24^. Curcumin activated caspase-9 (an intrinsic apoptotic factor)^25^ and caspase-3 in N2a cells, which are consistent with earlier studies^18^ but not caspases-8, an extrinsic apoptotic factor^26^. Proteolytic inactivation of PARP-1 (115 kDa) to 89 kDa fragment by activated caspase-3 is another hallmark of apoptosis^27^. Here we show for the first time that, curcumin significantly inhibited PARP-1 in N2a cells, which resembles earlier studies on breast cancers^28^ and glioma cells^29^. Therefore, curcumin-mediated N2a cells death confirmed intrinsic apoptotic pathway activation and making it a suitable neuroblastoma model to test the induction of endogenous *Bex* genes by a single agent, curcumin and to study their functions in neuroblastoma cell apoptosis.

Our results from curcumin treated N2a neuroblastoma cells indicated the induction of *Bex1, Bex2* and *Bex3* genes prior to activation of apoptotic pathways and continued to last for 24 hours post treatment. Adenoviral or trichostatin-A-mediated induction of *Bex1* and *Bex2* genes decreased glioma cells proliferation and tumor formation indicating their function as tumor suppressors in malignant glioma^12^. Re-expression of *Bex1* gene also functions as tumor suppressor in acute myeloid leukemia^30^ and pediatric intracranial ependymoma^31^. Similarly, *Bex3* has been shown to interact with p75NTR and mediates apoptosis^32^. Very recently, *Bex4* gene has been reported to be epigenetically silenced in oral squamous cell carcinoma (OSCC) and overexpression of *Bex4* reduced OSCC proliferation *in vitro* and subcutaneous tumor volume in *Bex4* overexpressing CAL27 mouse xenograft models *in vivo*^33^. However, there is no report on *Bex4* expression or function in the field of neuroblastoma. Furthermore, there is no report on the association of *Bex6* gene with any type of cancer cells death. In this study, we report for the first time that, *Bex4* and *Bex6* genes are re-expressed by curcumin and are associated with apoptotic N2a neuroblastoma cell deaths. Since Bex proteins are intrinsically disordered proteins, we also believe that these genes may not act in a similar manner in all cancer cell types and in normal cell processes^10^. In this study, we observed that all these genes were induced almost in a similar manner and remain overexpressed for a long period of time in response to curcumin-mediated N2a cells apoptotic death. Therefore, these results indicate us to interpret *Bex* genes as pro-apoptotic genes at least in N2a neuroblastoma cells. Furthermore, unlike curcumin, induction of *Bex* genes was not observed in MPTQ (a known anti-cancer agent)-mediated N2a cells death even at 25 μM dosage, known to induce DNA fragmentation and apoptosis approximately 3-fold more than curcumin^22^. In addition, tunicamycin, which is a well-known inducer of ER stress-mediated cytotoxicity in N2a cells^34^, also failed to induce *Bex* genes at cytotoxic dosage (0.1 μg/ml) in N2a cells. This observation not only eliminates the role of curcumin-induced ER stress on *Bex* genes induction in N2a cells but also strengthens the specific role of curcumin in *Bex* genes induction. Thus, curcumin is a specific chemical inducer of all the *Bex* genes and upregulation of these genes might be involved in N2a neuroblastoma apoptosis.

Next, we wanted to establish the mechanism behind curcumin-mediated induction of *Bex* genes. We utilized inhibitors of cell signaling, which are commonly used in experiments on cell physiology. In parallel with our decreased ERK1/2 phosphorylation prior to *Bex* genes induction, pretreatment with PD98059 and U0126 failed to reduce curcumin-mediated induction of *Bex* genes in N2a cells suggesting ERK1/2 activation is not required in *Bex* genes induction. In addition, reappearance of phosphorylation level of ERK1/2 at later stage suggests dephosphorylation of ERK1/2 might be an immediate early response to curcumin treatment in N2a cells. Curcumin induces reactive oxygen species (ROS) in human neuroblastoma cells^35^ and ROS generation can inhibit ERK1/2 phosphorylation in cancer cells^36^. It has also been reported that curcumin dephosphorylate ERK1/2 transiently up to 2 hours followed by reappearance of ERK1/2 phosphorylation by 6 and 12 hour of treatment in olfactory ensheathing cells^37^. In addition, curcumin induces ROS in mouse L929 cells within 5 minutes of treatment, which decreases to normal level by 60 minutes^38^. Therefore, inhibition of pERK1/2 phosphorylation at 15 min post curcumin treatment might be due to rapid generation of ROS, which might have decreased over time resulting in reappearance of phospho-ERK1/2 in N2a cells at later period of treatment. Furthermore, reduction in curcumin-mediated *Bex* gene induction was not seen in SB202190 pretreated N2a cells indicating p38 MAPK pathway is also not involved. However, wortmannin, SP600125 and pifithrin-α significantly inhibited curcumin-mediated induction of all the *Bex* genes in N2a cells. Wortmannin is a PI3-kinases inhibitor, which also inhibits PI3-kinase like kinases, such as ATM, ATR and DNA-PK^39^,^40^. ATM and DNA-PK phosphorylate p53 at ser15 in response to DNA damage, which is crucial for p53-dependent transactivation of downstream genes^41^,^42^. Therefore, inhibition of curcumin-mediated induction of *Bex* genes by wortmannin suggests the involvement of ATM or DNA-PK leading to p53 activation. JNK has been known to phosphorylate p53 at ser33 and ser15 in response to reactive oxygen species in breast cancer and colon carcinoma cell lines^43^. SP600125, an established JNK inhibitor has been shown to inhibit glutamate-induced p53-ser15 phosphorylation in murine neuronal hippocampal cell line^44^ suggesting SP600125-mediated inhibition of *Bex* genes induction in curcumin treated N2a cells might involves JNK-mediated p53 activation. Pifithrin-α has been shown to inhibit DNA binding activity of p53 without affecting its phosphorylation status^23^. Results from cell signaling studies show pifithrin-α is the most potent inhibitor of curcumin-mediated induction of all the *Bex* genes, suggesting the involvement of p53 activation in curcumin-mediated induction of *Bex* genes. Results from p53 activation indicated a significant reduction in p53 level by 60 minutes in curcumin treated N2a cells than untreated controls. Increased p53-ser15 phosphorylation but decreased p53 level have been reported in acetaminophen treated glioma cells, which has been linked with ubiquitin-dependent degradation of p53^45^. Similarly, Kuenzi *et al*., have reported increased p53-ser15 phosphorylation but decreased expression of p53 in fungal alkaloid, militarinone A-treated N2a cells^46^, indicating the activation of p53 by increased phosphorylation at ser15 might reduce p53 level in curcumin treated N2a cells. In addition, our results also indicated p53-ser15 hyperphosphorylation immediately (15–30 minutes) after curcumin treatment but before *Bex* genes induction in neuroblastoma cells.

Furthermore, to establish p53 as a direct transcriptional activator of *Bex* genes, the promoters of *Bex* genes were screened manually for p53 binding elements as reported by Deiry El *et al*.^47^ (5′-RRRCWWGYYY (0–13 bases) RRRCWWGYYY-3′) and Veprintsev *et al*.^48^ (5′-NDRCATGYYY (0–13 bases) NDRCATGYYY-3′or 5′-NNDCWWGYHN (0–13 bases) NNDCWWGYHN-3′)^47^,^48^. DNA binding elements of p53 were found in the promoter regions of *Bex1, Bex2, Bex3, Bex4* and *Bex6* genes as shown in Supplementary Fig. S5. Our results from chromatin immunoprecipitation utilizing anti-phospho-p53-ser15 antibody for the first time demonstrate that all the *Bex* genes have at least one functional p53 binding elements in their promoters and p53 can transactivate all the *Bex* genes in response to curcumin treatment in N2a cells. As suggested earlier, inhibitors of curcumin-mediated induction of *Bex* genes, wortmannin, SP600125 and pifithrin-α also reduced p53-ser15 phosphorylation significantly indicating the involvement of PI3-kinase like kinases and JNKs in activating p53, which might be associated with curcumin-mediated induction of *Bex* genes in N2a neuroblastoma cells. Interestingly, the time of p53 activation paralleled with the time of ERK1/2 inactivation. Inhibition of ERK1/2 stimulated p53 activation in MCF7 cells that led to the upregulation of downstream targets, like p21 and Bax^49^ and activation of p53 stimulate intrinsic apoptotic pathway^50^,^51^. In addition, wortmannin, SP600125 and pifithrin-α reduced curcumin-mediated binding of phospho-p53-ser15 onto p53 DNA binding elements in *Bex* gene promoters. Collectively, our results for the first time provide a strong evidence for presence of functional p53 DNA binding elements in all *Bex* genes, which can be epigenetically activated by curcumin.

Finally, to link the function of *Bex* genes induction with neuroblastoma cell apoptosis, experiments involving reduction of Bex mRNAs either by siRNA or pifithrin-α (most potent inhibitor of *Bex* gene induction) treatment along with reduction of curcumin-mediated N2a cell deaths were performed. Our results clearly demonstrated, both pifithrin-α and *Bex1, 2, 4* and *6* gene siRNAs either individually or in combination demonstrated significant reduction in curcumin-mediated cell death in N2a cells. Although the use of siRNA against *Bex* genes are limited but a recent report suggests siRNA-mediated inhibition of only *Bex4* gene promotes proliferation of OSCC cells^33^ suggesting the tumor suppressor activity of *Bex4* in OSCC cells. Pifithrin-α also significantly inhibited proteolytic activation of apoptotic factors such as caspase-9, caspase-3 and inactivation of PARP-1, providing a strong evidence for a functional association of *Bex* genes in neuroblastoma cell apoptosis mediated by curcumin.

**Figure 8:**
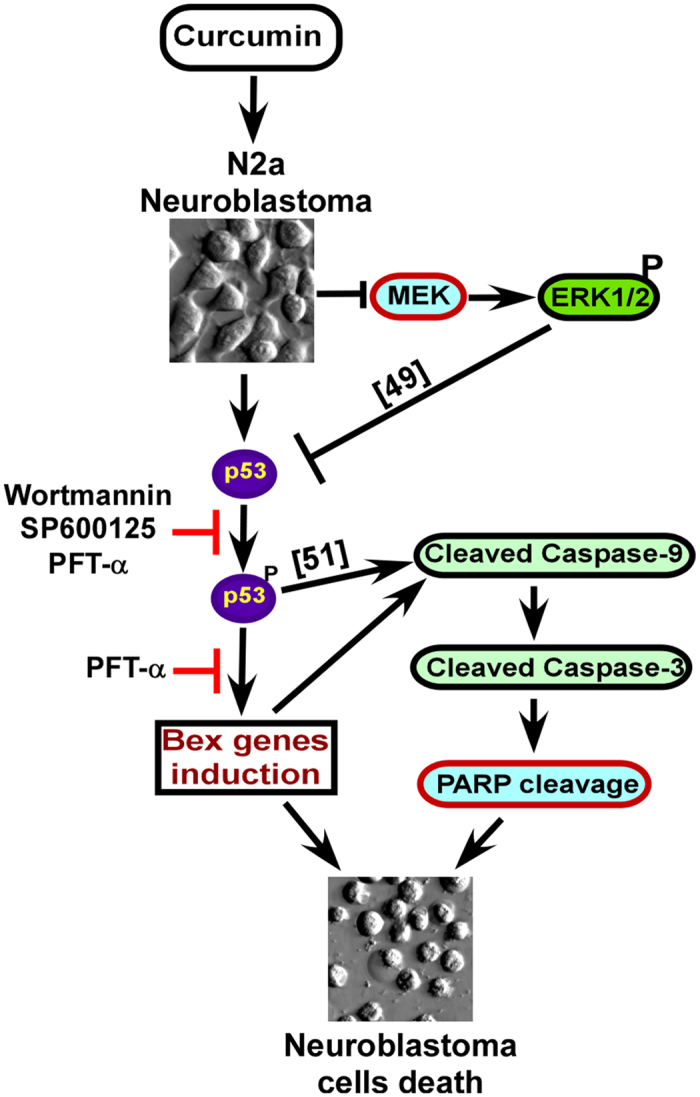
Schematic representation of mechanisms behind curcumin-mediated *Bex* genes induction and apoptosis in neuroblastoma cells. Curcumin induces *Bex* genes prior to apoptotic N2a cells death. Wortmannin, SP600125 and pifithrin-α abrogate curcumin-mediated induction of *Bex* genes suggesting the involvement of PI3-kinases, JNKs and p53respectively. Curcumin activated p53 phosphorylation at ser15 and inactivated MEKs at a similar time but well before the induction of *Bex* genes. Phosphorylation of p53-ser15 is inhibited by wortmannin, SP600125 and pifithrin-α indicating a molecular association of p53 activation with curcumin-mediated *Bex* genes induction. Furthermore, inhibition of *Bex* genes by pifithrin-α also inhibited curcumin-mediated N2a cell deaths suggesting a strong correlation of *Bex* genes induction with apoptotic neuroblastoma cells death.

In conclusion, the present study indicates for the first time the induction of all *Bex* genes by ectopic application of curcumin, a commonly used phytochemical. Moreover, curcumin is a specific inducer of *Bex* genes unlike MPTQ and tunicamycin. Cell signaling studies for the first time demonstrate the association of p53, PI3-Kinase and JNK in curcumin-mediated induction of endogenous *Bex* genes and the activation of intrinsic apoptotic pathway (Fig. 8). This study for the first time also reports, *Bex* genes are novel downstream targets of p53 and possess active p53 DNA binding elements in the promoter region of all *Bex* genes. In addition, based on the experimental evidence, induction of *Bex* genes are associated with curcumin-mediated apoptosis in N2a neuroblastoma cells. Therefore, induction of *Bex* genes (especially *Bex6, Bex1* and *Bex4* genes) functions as tumor suppressors at least in N2a neuroblastoma cells and may serve as an alternative for the treatment of neuroblastomas and other cancers with silenced *Bex* genes.

## Material and Methods

### Cell culture

Mouse neuroblastoma cell line N2a (CCL-131; ATCC, USA) was procured from Prof. Nihar Ranjan Jana, National Brain Research Center (NBRC), Manesar, India^18^. Cells were grown in Dulbecco Modified Eagle’s Medium (DMEM) (Sigma-Aldrich, USA and Invitrogen, USA) containing 5% heat inactivated fetal bovine serum (FBS) (Hyclone, USA), 100 U/ml penicillin and 100 mg/ml streptomycin (Life Technologies, USA) in a humidified incubator at 37 °C with 5% CO_2_.

### Preparation of reagents

Curcumin (Sigma) stock solutions of 30, 75 and 150 mM were prepared in cell culture grade dimethylsulfoxide (DMSO). MPTQ (8-methoxypyrimido[4′,5′:4,5]-thieno(2,3-b)quinoline-4(3H)-one), an anti-cancer agent^22^ was procured from Dr. Sathees C. Raghavan, IISc, Bangalore, India. MPTQ stock solutions of 7.5, 15, 30 and 75 mM were prepared in DMSO. Tunicamycin (Sigma) stock solution of 0.3 mg/ml was also prepared in DMSO. Wortmannin and SP600125 (Tocris) stock solutions of 12 μM and 900 μM respectively were prepared in DMSO. Pifithrin-α (Sigma) stock solution of 50 mM was also prepared in DMSO. These stock solutions were diluted to final concentrations in serum free DMEM just before treatment.

### Antibodies

Anti-caspase-3, anti-caspase-9, anti-phospho-p53-ser15 and anti-p53 antibodies were purchased from Cell Signaling Technology Inc., USA. Anti-PARP-1, anti-phospho ERK1/2, anti-ERK1/2 and anti-GAPDH antibodies were purchased from Santa Cruz Biotechnology, USA. Anti-cleaved caspase-3 antibody was purchased from Imgenex Corp., USA. Anti-caspase-8 antibody was purchased from R&D systems, Inc., USA. Horseradish peroxidase (HRP)-conjugated secondary antibodies and Alexa-Fluor 647 conjugated F(ab’)^2^ fragment antibodies were purchased from Thermo-Pierce and Molecular probes respectively.

### Bright Field Imaging

N2a cells were seeded at a density of 20000 cells/well in 24 well plates. After 2 days, cells were serum starved for 2 hours followed by treatment with 50 μM of curcumin or equivalent amount of DMSO as controls. After 24 hours of treatment, bright field images of cells were captured using 40X objective lens with Hoffman modulation and thickness correction rings in Nikon TS100 inverted microscope supported by NIS elements BR (2.3 version) software.

### LIVE/DEAD Assay

N2a cells were seeded at a density of 5000 cells/well in 96 well plates. Curcumin-mediated N2a cell death was studied using LIVE/DEAD assay reagents (Invitrogen) as described earlier^22^. Briefly, cells were serum starved for 2 hours and treated with 10, 25, 50 μM of curcumin or equal volume of DMSO as control for 24 hours. Cells were then incubated in media containing calcein-AM and ethidium homodimer for 30 minutes at room temperature in dark. Random images (3–4) were captured using 40X objective lens in Nikon TS100 inverted microscope supported by NIS elements BR (2.3 version) software with same exposure settings. Number of live (green) and dead (red) cells were counted using ImageJ software.

### MTT Assay

MTT assay was performed as described earlier^52^. Briefly, 5000 cells/well were cultured in a 96-well plate for 72 hours. Cells were treated with indicated concentration of curcumin after 2 hours of incubation in serum free DMEM. After 24 hours, the media was completely replaced with serum free DMEM containing 1 mg/ml of MTT reagent (Sigma) and cultured for another 4 hours. The media containing MTT was completely removed and intracellular formazan products were dissolved in MTT solvent (4 mM HCl and 0.1% NP-40 in isopropanol) for 15 minutes with shaking. The absorbance of the samples was measured at 570 nm using a multi-well plate reader.

### DNA Fragmentation

N2a cells were seeded at a density of 24,000 cells/well in 24-well plates for 72 hours. Cells were then treated with varying amount of curcumin for 24 hours or treated with equal volume of DMSO as controls after 2 hours of incubation in serum free DMEM. Cells were then lysed using 100 μl of digestion buffer (100 mM NaCl, 10 mM Tris-Cl pH 8.0, 1 mM EDTA (pH 8.0), 0.1% SDS and 0.1 mg/ml proteinase-K) at 50 °C for 3 hours. Samples were mixed with 5 μl of SDS-OUT reagent and incubated on ice for 20 minutes followed by centrifugation at 10,000 × g for 10 minutes at 4 °C. Supernatants were collected and treated with RNase for 20 minutes on ice. After DNA quantification, 2.5 μg of DNA from each sample was resolved in ethidium bromide stained 1.5% agarose gels and imaged in a gel-doc system. Integrated density of fragmented DNA normalized with total DNA intensity was measured and fold change was calculated.

### TUNEL Assay

N2a cells were seeded at a density of 0.5 × 10^6^ cells per 25 cm^2^ flasks and cultured for 2 days. Cells were then incubated in serum free DMEM for 2 hours and treated with indicated amounts of curcumin or equal volume of DMSO as control. After 24 hours, TUNEL assay was performed in suspension as described earlier^22^. Labeled cells were spotted on slides coated with tissue adhering solution (Cancer diagnostics) and mounted using anti-fade gold mounting media containing DAPI (Invitrogen). Fluorescent images were captured using 40X lens using similar exposure settings in a Nikon Ti eclipse microscope supported by Metamorph software (version 7.7.0.0). TUNEL positive cells were counted and percent positive cells were plotted as histograms.

### RNA Isolation

N2a cells treated with varying amount of curcumin for 2 hours or with 25 μM of curcumin for indicated time points after 2 hours of serum starvation. Equivalent amount of DMSO treated cells were used as controls. Similar approach was adopted for N2a cells treated with different dosages of MPTQ for 4 hours. For inhibitor assay, N2a cells were pretreated with 4 nM of wortmannin, 300 nM of SP600125 and 25 μM of pifithrin-α for 30 minutes followed by 25 μM of curcumin treatment for 4 hours. DMSO treated and only curcumin treated N2a was used as negative and positive controls of the experiment respectively. Total RNA was isolated using Trizol (Invitrogen) as per manufacturer’s instructions and quantified by utilizing Nanovue spectrophotometer (GE healthcare). Total RNA was treated with 2 units of amplification grade DNase I (Life technologies, USA) for 15 minutes at room temperature. The DNase activity was inhibited using EDTA to a final concentration of 2 mM and incubated at 65 °C for 10 minutes. DNA free RNA was further quantified by Nanovue spectrophotometer.

### Reverse Transcription and Polymerase Chain Reaction (RT-PCR)

Superscript III RT PCR kit (Invitrogen) was used to synthesize cDNA from 5 μg of DNA free RNA. The RT products were diluted 10-fold before PCR. The following primers (Sigma) were used to amplify Bex mRNA. *Bex1*, forward: 5′-AAGGCGTGAAAAATCTCAACA-3′, reverse: 5′-GCATGAGGCAAAA CTCATCA-3′. *Bex2*, forward: 5′-GAAGAAAAGCCACAGGATGC-3′, reverse: 5′-CAGGGCA TAAGGCAAAACTC-3′. *Bex3*, forward: 5′-GTCCACCAGGAAAACGAAGA-3′, reverse: 5′-ATCGGAAGTTAGGGGCAAGT-3′. *Bex4*, forward: 5′-TGAAGAAGAACCCCACCATC-3′, reverse: 5′-TTGCCATGCTAACAGGAATG-3′. *Bex*6, forward: 5′-TGATGTCCAAAGTCAAACAAGT-3′, reverse: 5′-TGTGAGGAACCACTTGGAAA-3′. GAPDH, forward: 5′-AACTTTGGCATTGTGG AAGG-3′, reverse: 5′-TGTGAGGGAGATGCTCAGTG-3′ and β-actin, forward: 5′-TGTTACCA ACTGGGACGACA-3′, reverse: 5′-GGGGTGTTGAAGGTCTCAAA-3′. PCR amplification of desired genes was performed using diluted RT products and G0 Taq polymerase (Promega, USA) for either 32, 34 or 36 cycles. The PCR products were separated using 2% agarose gel electrophoresis. Images were captured using a ChemiDoc XRS^+^ gel doc system (Bio-Rad). Densitometric analysis was done using Image Lab (version 3.0) software and fold change between untreated and treated samples was calculated after normalization with GAPDH or β-actin PCR products.

### Western blotting

Western blot analysis was performed using earlier protocol^53^. Briefly, cells were collected after the treatments and centrifuged at 2000 rpm for 5 minutes to obtain cell pellet. Supernatant was removed completely and cell lysates were prepared in lysis buffer. Lysates were sonicated, cleared by centrifugation at 500 × g for 5 minutes at 4 °C, aliquoted and stored at −70 °C deep freezer. Protein content of each lysate was estimated by bicinchoninic acid assay using micro BCA protein estimation kit (Thermo-Pierce). Equal amount of proteins (~60 μg) from each sample were separated by SDS-PAGE and transferred onto nitrocellulose or PVDF membranes. After blocking, membranes were blotted with primary antibodies at 4 °C for overnight. Horseradish peroxidase conjugated secondary antibodies were used to develop blots. Bands were detected using West Pico supersignal chemiluminiscent substrate (Pierce). GAPDH immunoblotting was used to normalize protein loading and transfer between the samples.

### Immunocytochemistry

N2a cells were plated at a density of 10,000 cells/well onto Poly-D-Lysine (0.05 mg/ml) coated coverslips placed in 24 well plates. After 72 hours, cells were serum starved for 2 hours and treated with 25 μM of curcumin or with equal amount of DMSO (controls) for 30 minutes. Cells were treated with pharmacological inhibitors 30 minutes prior to curcumin treatment. Cells were quickly washed with 1X PBS and fixed with 4% paraformaldehyde for 30 minutes at RT. Cells were then permeabilized with 0.3% triton X-100 followed by blocking (PBS with 0.1% BSA and 10% normal goat serum) for 1 hour at RT. Cells were incubated with primary antibodies in blocking buffer at 4 °C overnight in a humidified chamber. After several washes, cells were incubated with appropriate secondary antibody conjugated with Alexa Fluor 647 for 1 hr at RT. Cells were washed several times and mounted with anti-fade mounting medium containing DAPI. Fluorescent images were captured using 40X plan Fluor lens in Nikon Ti eclipse microscope and at same exposure settings^54^. Fluorescence intensity was measured by multi-wavelength cell scoring module of Metamorph software (version 7.7.0.0) with identical settings. Images were displayed with same image scale using Metamorph software.

### Synthesis of small inhibitory RNA duplexes for Bex mRNAs

The small inhibitory RNA duplexes for Bex mRNA were designed and synthesized as described earlier^55^,^56^. Briefly, 21-nucleotide sequences of Bex siRNAs were synthesized with following sequences: sense, Bex1: 5′-GAG GTC AGG GCC ACA GCT GGC TAT AGT GAG TCG TAT TA-3′, antisense Bex1: 5′-GAG CCA GCT GTG GCC CTG ACC TAT AGT GAG TCG TAT TA-3′, sense Bex2: 5′-AGG CAC GTA GTA GTC TCC AGC TAT AGT GAG TCG TAT TA-3′, antisense Bex2: 5′-GAGCTG GAG ACT ACT ACG TGC TAT AGT GAG TCG TAT TA-3′, sense Bex4: 5′-ATG GCC CAG AGA AAG TTA GGC TAT AGT GAG TCG TAT TA-3′, antisense Bex4: 5′-GTG CCT AAC TTT CTC TGG GCC TAT AGT GAG TCG TAT TA-3′, sense Bex6: 5′-CTG CCC TAC AAA TCT CCC AGC TAT AGT GAG TCG TAT TA-3′, antisense Bex6: 5′-ATG CTG GGA GAT TTG TAG GGC TAT AGT GAG TCG TAT TA-3′ containing T7 promoter sequence, which is underlined. Similarly, the scrambled siRNA was synthesized with the following sequences: sense scrambled: 5′-CAG TGA GGT CAC GAT CCG ATC TAT AGT GAG TCG TAT TA-3′, antisense scrambled: 5′-GTG ATC GGA TCG TGA CCT CAC TAT AGT GAG TCG TAT TA-3′. Equal nano mole of siRNA and T7 oligonucleotides were annealed, and 200 pmol of annealed oligonucleotides were used to synthesize 21-oligonucleotide long RNA using Riboprobe *in vitro* transcription kit (Promega) as per manufacturer’s instructions. The contents of sense and antisense transcripts were mixed and annealed to obtain RNA duplexes as final siRNA construct for *Bex* genes. The precipitated RNA was pelleted, washed with 70% ethanol and dissolved in 50 μl of RNase free water. The RNA was quantified using Nano spectrophotometer and used for transfection experiments.

### Transfection of siRNA duplex into N2a neuroblastoma cells

N2a cells were transfected with 200 pico moles of Bex siRNAs either individually or all four (Bex1, Bex2, Bex4 and Bex6) in combination at 30–40% confluency using lipofectamine 2000 (Invitrogen, USA) reagent. The media was replaced after 24 hours of transfection. At 72 hours of transfection, N2a cells were serum starved for 2 hours followed by curcumin (25 μM) treatment. Control cells were treated with equivalent amount of DMSO. After 24 hours, LIVE/DEAD assay was performed to establish the relation between *Bex* gene induction and cell death. Fluorescent images were captured from nine random fields per sample using 20X objective lens in Nikon Ti eclipse microscope and at same exposure settings. Number of ethidium homodimer positive (dead and red) cells were counted per field, analyzed and plotted as histograms. Fluorescent images from 4X objective lens were also obtained to cover more area and from the center of the well where dead cells have a tendency to accumulate. The intensity of ethidium homodimer (indicator of dead cell) was measured from 4X magnified image by utilizing Metamorph software and analyzed for graphical presentation.

### Chromatin Immunoprecipitation

Approximately 70–80% confluent N2a cells were serum starved for 2 hours followed by treatment with 25 μM of curcumin or with equal amount of DMSO (controls) for 30 minutes. For inhibitor assay, cells were pretreated with 4 nM of wortmannin, 300 nM of SP600125 and 25 μM of pifithrin-α followed by 25 μM of curcumin treatment for 30 minutes. Chromatin immunoprecipitation was performed as reported earlier^57^. Briefly, cells were cross-linked with 1% paraformaldehyde for 10 min at room temperature. Cells were washed with 1X PBS twice and cell lysates were prepared in lysis buffer (50 mM Tris-Cl pH 8.0, 1% SDS, 10 mM EDTA, 1X protease inhibitor cocktail and 1X phosphatase inhibitor cocktail) for 30 minutes on ice. Lysates were sonicated and supernatants were recovered by centrifugation at 12000 rpm for 10 minutes at 4 °C. Concentration of protein in each lysate was measured as described earlier and 250 μg of protein equivalent lysate was diluted to 1 ml using dilution buffer (150 mM NaCl, 20 mM Tris-Cl pH 8.0, 2 mM EDTA and 1% Triton X-100). The diluted lysates were precleared and immunoprecipitated with 5 μl of anti-phospho-p53-ser15 antibody, 2 μg of sheared salmon sperm DNA and 20 μl of Protein A-sepharose beads at 4 °C overnight. After several washings in dilution buffer at 4 °C, the antibody, antigen and DNA complex was eluted in elution buffer (1% SDS and 0.1 M NaHCO_3_). DNA protein cross-linking was reversed in ChIP and input samples by heating overnight at 65 °C with 0.3 M NaCl. The protein was digested using proteinase K (10 μg/ml) for 1 hr at 55 °C. DNA extraction was done by phenol-chloroform-isoamyl alcohol (25:24:1), precipitated at −20 °C using 0.1 volume of sodium acetate and 2.5 volume of ethanol, washed with chilled 70% ethanol, air dried and dissolved in 20 μl of nuclease free water. PCR amplification was done using 1 μl of DNA with primers flanking p53 binding site at −2000 to −2023 bp of *Bex1* promoter (Forward primer: 5′-TGAACACTACGCATCCTATG-3′, Reverse primer: 5′-CATCTAGAACACACTCCTCT-3′), at −2462 to −2493 bp of *Bex2* promoter (Forward primer: 5′-TCTGTATGCATCCACTGTAG-3′, Reverse primer: 5′-CACTTCTGAAACTGTTCCACA-3′), at −1883 to −1914 bp of *Bex*3 promoter (Forward primer: 5′-CTCAGGCGACTCTGGTTT-3′, Reverse primer: 5′-TCAACATGGCCAAGCCAC-3′), at −4462 to −4486 bp of *Bex*3 promoter (Forward primer: 5′-GCAGGAGCTGATGCAGAA-3′, Reverse primer: 5′-CTCCATGAGATCCAGCTG-3′), at −912 to −943 bp of *Bex*4 promoter (Forward primer: 5′-CCTTCACTTTCCGTGCCT-3′, Reverse primer: 5′-CCCCTACCTCAATAAAAAG-3′), at −1956 to −1977 of *Bex*4 promoter (3′Forward primer: 5′-CACTGATCTTCAGAGTCTCCA-3′, Reverse primer: 5′-CTTGCTTTCTGTTTTCCTCT-3′), at −2484 to −2509 bp of *Bex4* promoter (Forward primer: 5′-ACTATTAGAAGGTATGGCCC-3′, Reverse primer: 5′-GAGGTTCAGTCCATTATCATC-3′) and at −458 to −482 bp of *Bex6* promoter (Forward primer: 5′-GACCTGGAAACACCTAGA-3′, Reverse primer: 5′-GCACTGTCTGTCTAATGA-3′).

### Statistical Analysis

At least three independent experiments or data points were used for analysis. Values obtained after analysis were expressed as histograms of mean ± SD or SEM. Statistical analysis was performed using Microsoft Excel and Sigmastat 3.5 softwares. Comparison between two groups and multiple groups were performed using two-tailed unpaired Student’s t-test and One-way ANOVA respectively. Non-parametric one-way ANOVA was performed if the values failed normality or equal variance test. Differences between groups were considered statistically significant when p ≤ 0.05.

## Additional Information

**How to cite this article**: Sidhar, H. and Giri, R. K. Induction of *Bex* genes by curcumin is associated with apoptosis and activation of p53 in N2a neuroblastoma cells. *Sci. Rep.* **7**, 41420; doi: 10.1038/srep41420 (2017).

**Publisher's note:** Springer Nature remains neutral with regard to jurisdictional claims in published maps and institutional affiliations.

## Supplementary Material

Supplementary Information
