# Supplementary material for: Induction of Bex genes by curcumin is associated with apoptosis and activation of p53 in N2a neuroblastoma cells
Source: Sci Rep. 2017 Feb 1;7:41420. doi: 10.1038/srep41420 (PMC5286441; doi:10.1038/srep41420)
Supplement: Supplementary Information [file srep41420-s1.pdf]

Induction of *Bex* genes by curcumin is associated with apoptosis and activation of p53 in N2a neuroblastoma cells

Himakshi Sidhar[1] and Ranjit K. Giri[1*]

[1]National Brain Research Centre, Manesar, Haryana, India 122051

***Corresponding author:**

Cellular and Molecular Neuroscience Division, National Brain Research Centre, Manesar, Haryana, 122051, India.

Telephone: 91-124-2845216

Fax: 91-124-2338910

E-mail: ranjit@nbrc.ac.in

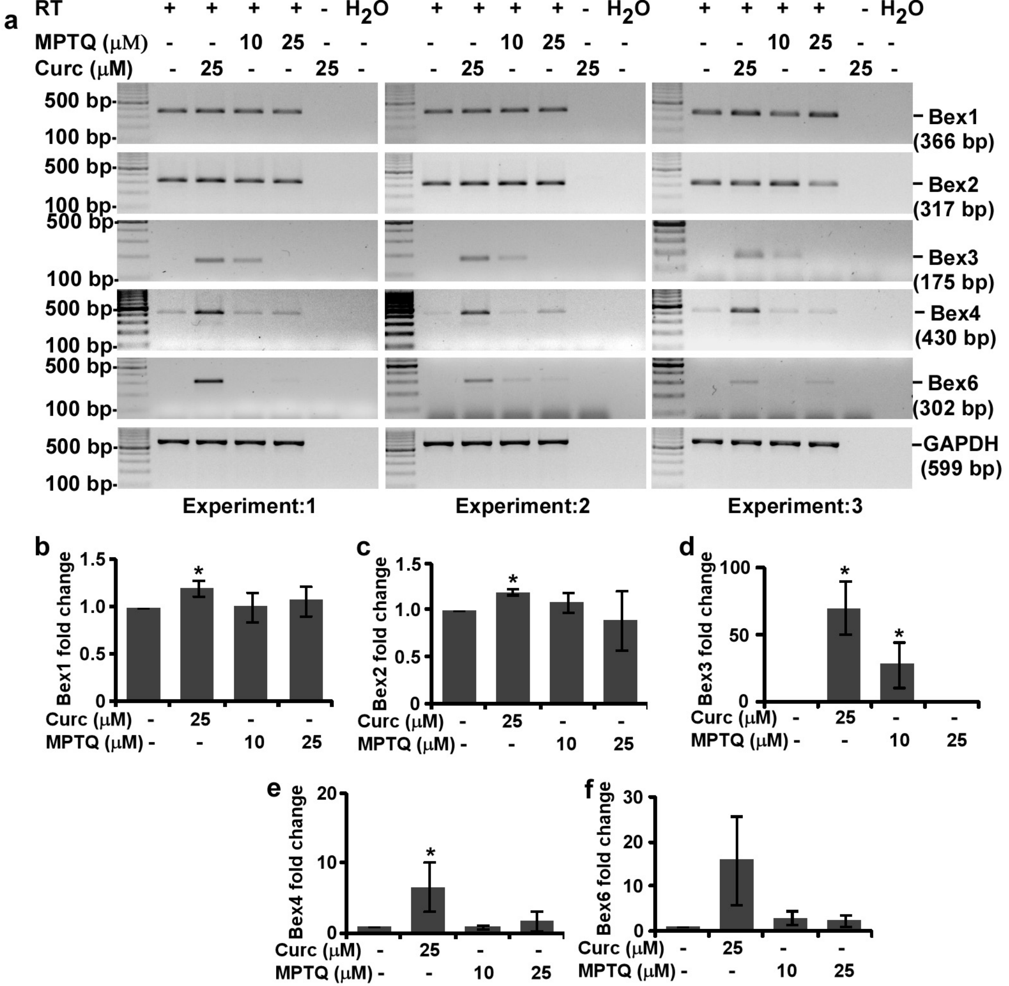

Supplementary Fig. 1. Curcumin specifically induces all the *Bex* genes in N2a cells. (a) N2a cells were cultured, serum starved for 2 hours and treated with indicated concentrations of either MPTQ (10, 25 µM) or curcumin (25 µM). DMSO treated N2a cells were used as controls. After 4 hours of treatment, total RNA was isolated and expression of all the Bex mRNAs was studied by RT-PCR analysis. GAPDH was used as loading control. Densitometric analysis of Bex1 (**b**), Bex2 (**c**), Bex3 (**d**), Bex4 (**e**) and Bex6 (**f**) PCR products was performed and normalized with corresponding GAPDH band intensity. Values are displayed as histograms of mean±standard deviation from three independent experiments. p values displayed were calculated by using two-tailed, unpaired Student's t-test and *=$p \leq 0.05$ is considered statistically significant.

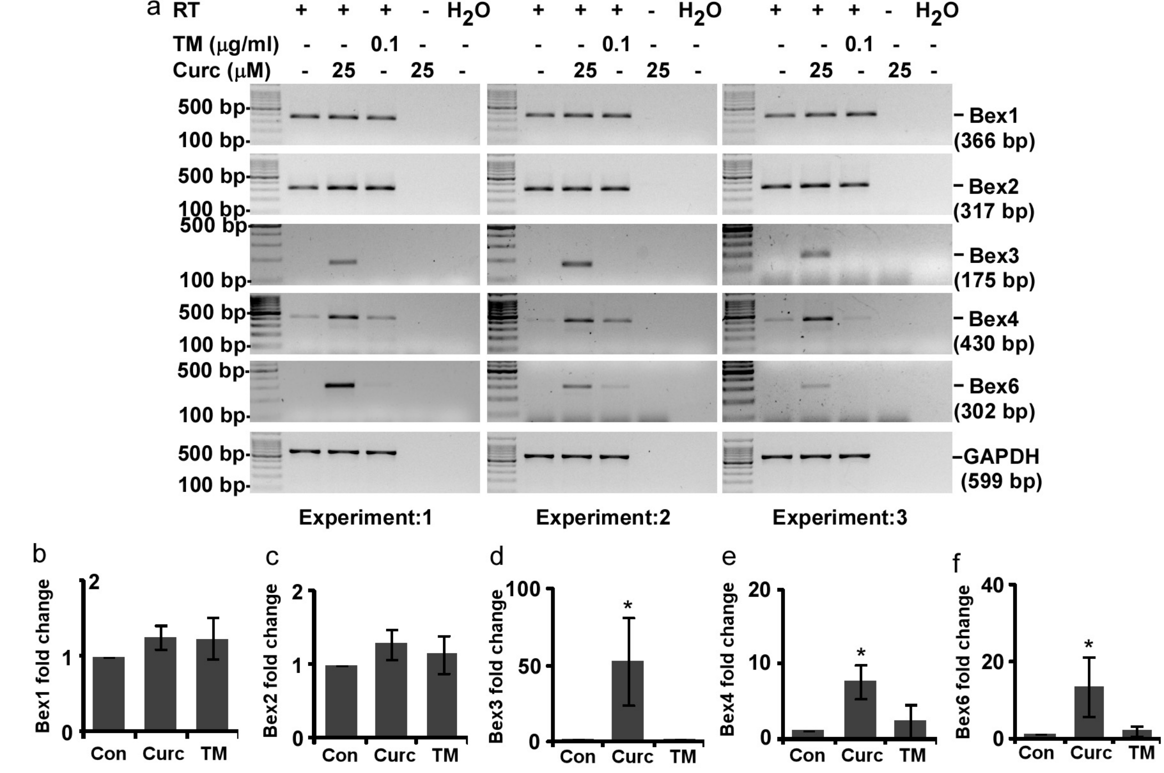

Supplementary Fig. S2. Curcumin-mediated induction of *Bex* genes is not associated with endoplasmic reticulum (ER) stress involved in N2a cells. (**a**) Approximately 70% confluent N2a cells were serum starved for 2 hours and then treated with either ER stress inducer tunicamycin (0.1 µg/ml) or curcumin (25 µM). Control cells were treated with equivalent amount of DMSO. Expression of all the Bex mRNAs was studied after 4 hours of treatment by RT-PCR analysis. GAPDH was used as loading control. Densitometric analysis of Bex1 (**b**), Bex2 (**c**), Bex3 (**d**), Bex4 (**e**) and Bex6 (**f**) PCR products was performed and normalized with corresponding GAPDH band intensity. Values are displayed as histograms of mean±standard deviation from three independent experiments. p values displayed were calculated by two-tailed, unpaired Student's t-test and *=p≤0.05 is considered statistically significant.

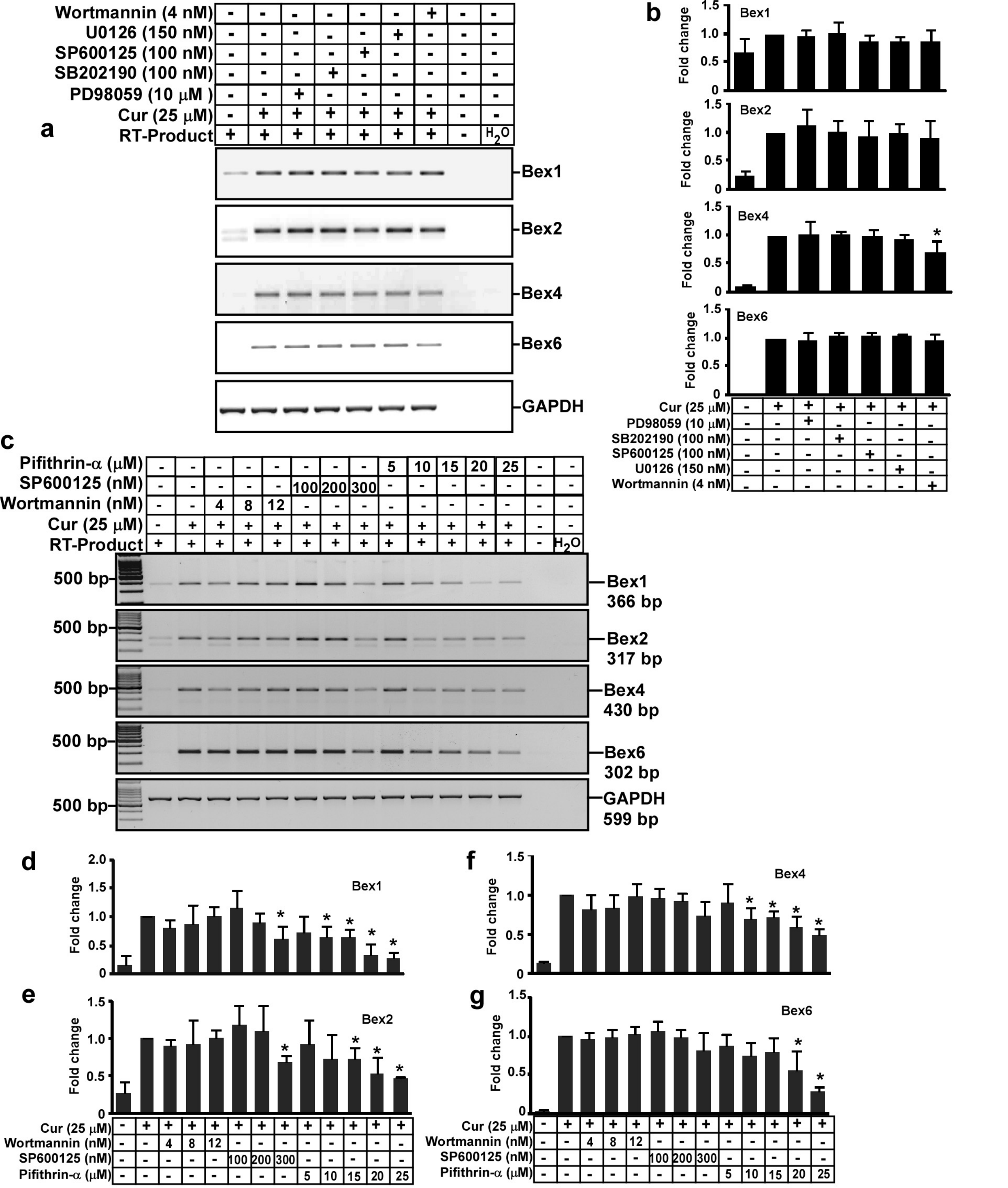

Supplementary Fig. S3. Screening of cell signaling pathways involved in curcumin-mediated induction of *Bex* genes utilizing pharmacological inhibitors in N2a neuroblastoma cells. Our results demonstrate curcumin-mediated induction of *Bex* genes are regulated by PI3-Kinase, JNK and p53 activation.

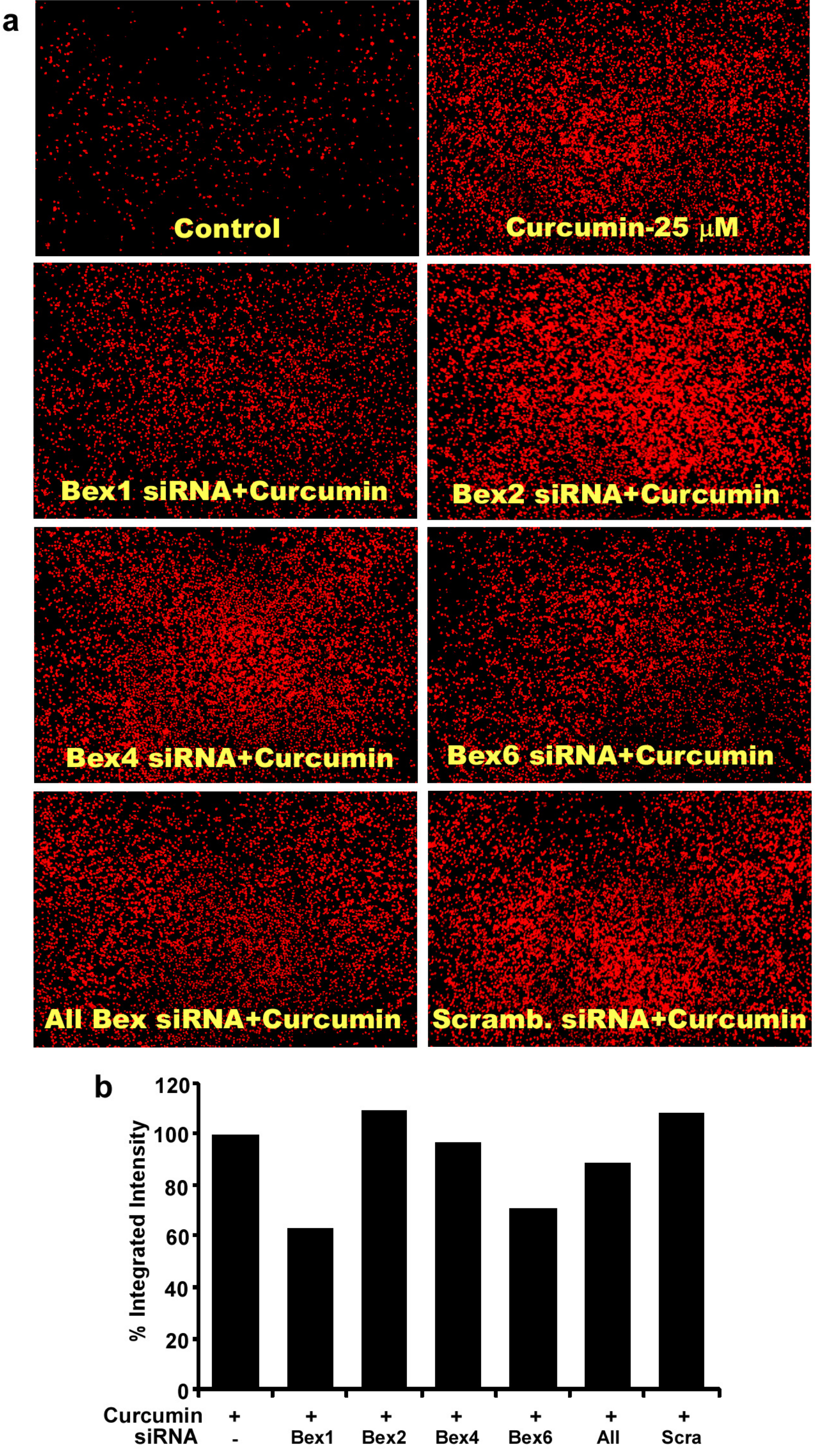

Supplementary Fig. S4. Association of *Bex* genes with curcumin-mediated N2a cell apoptosis. Transfection of N2a cells with siRNA for various Bex transcripts exhibit maximum inhibition of curcumin-mediated N2a cell apoptosis by siRNA for Bex1 and Bex6 followed by combination of all siRNA and Bex4 employing Live-Dead assay coupled with low magnification (4X objective lens) imaging. The level of cell deaths in cells transfected with scrambled siRNA is comparable to curcumin-mediated N2a cell apoptosis.

a p53 DNA binding element (El-Deiry et al., 1992) 5' RRRCWWGYYY (0-13 bases) RRRCWWGYYY

p53 DNA binding element (Veprintsev et al., 2008) 5' NDRCATGYYY (0-13 bases) NDRCATGYYY
or
5' NNDCWWGYHN (0-13 bases)NNDCWWGYHN

b

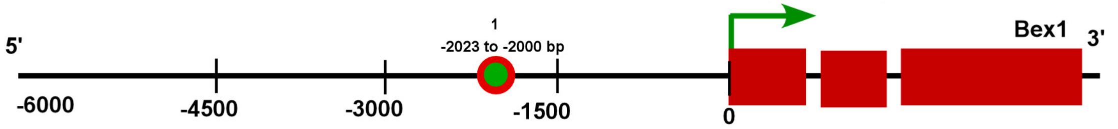

(-2136 from TSS) 5'-
TGAACACTACGCATCCTATGGAATCATAACATGAAGCCAGACCACATTTTCTGGGAGTAGGATAGTAAGTGCAGGATTGTG
ATATAGAAACTAATGTTACTAATCAAAGGAAATAACAAGCTACTCACACAAGCAATGACAGATGAATCTCA AAAGATATGCTT
AGGGAAAGCTACCAGGTAAAGAGGAGTGTGTTCTAGATG-3' (-1933 from TSS)

c

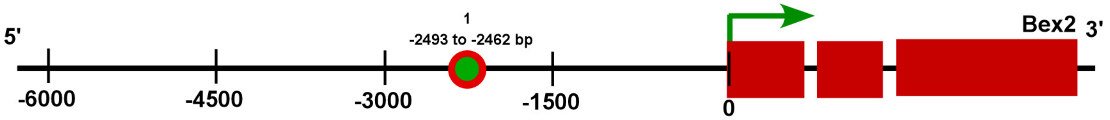

(-2569 from TSS) 5'-
TCTGTATGCATCCACTGTAGCTTGGTCAGTCTACCAGAAGCCAGTAACTGTTCAGCCCTTACACGTGAATGCACCCA
ATCAT GTTGGATGAGAAATTTTGCTTGTATTTTCACCAGGGAAATTGGCTTGTAGCTTTCTTTTTCTGTTATGTCT
TTTATCCAGATTTGTTGTCATAATACTAATTGGCTTCATAGAATTAGCTTTCTCGTGTTCCTTCTGTTTTTACTTTGTGGAAC
AGTTTCAGAAGTG -3' (-2320 from TSS)

d

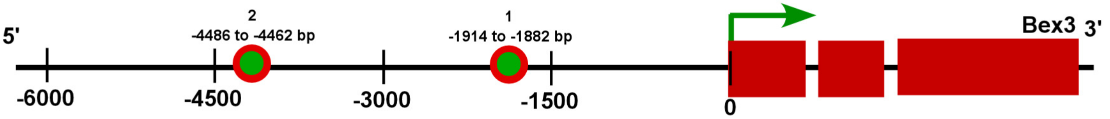

(-1983 from TSS) 5'-
CTCAGGCGACTCTGGTTTGTATCAAGTTGACAAAGAACTTACCAATGTACTATGGTTTATTTTTTTTAAAGGCAAGCAGTAAG
CTGTAATTAATCAAGCTGTCACCCAACTTCAGTTCCGTTTTCTGTACATTTCCATTTTCTGTCCAAAACATGTGGCTTGGCCAT
GTTGA-3' (-1811 from TSS)

(-4526 from TSS) 5'-
GCAGGAGCTGATGCAGAAGCCATGGAGGGATGCTCCTTACTGGCTTGCTTCCACTGGCTTGCTCAGCCTGCTCTCTTATA
AAACCCAGGACTACCAGTCTAGGGATGGCACCACCCACAATGGACCCTCCCACCCTTGATTCTTACAGCTGGA TCT
CATGGAG-3' (-4364 from TSS)

e

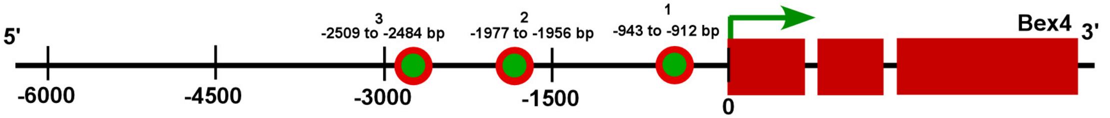

(-978 from TSS) 5'-
CCTTCACTTTCCGTGCCTGTGGTGTACTCATGTGTATGCATGTTGATACACATGTGTATGCATGTTGATACACATGTGCCTAT
GTAAATCATCTTCCAATGCTCTTCTACCTTTTTATTGAGGTAGGGG-3' (-849 from TSS)

(-2071 from TSS) 5'-
CACTGATCTTCAGAGTCTCCAGGTGGTGGTAACTAAGGTTAAGCTCCAGTTAAGCTTCATTATTCCTTCTCCATCCCT
TTGCTTTTCTCCACTTTAACATGTAAAGGTCATGTCCATAAACAAGGC TCTTTTTCTAGGGAGCCAAAC
CTAAAAAAGGAAGTCCTATGTCAATAGTCCA ACTAAAATTACAAAAAAGAGGAAAACAGAAAGCAAG-3'(-1857 from TSS)

(-2605 bp from TSS) 5'-
ACTATTAGAAGGTATGGCCCTGTTGGAGTAGGTGTAGCCTTTTTGGAGTAGGTGTGTCACTGTGGATGAGGGCTTTAAGAC
CCTCATCCTAGCTATCTGCAAGTCAATATTCTGCTAGCAGCCTTCAGATGAAGACGTAGAACTCTCATCTC
CGCCAGCACCATGCCTGCCTGGATACTGCCATGCTCCCACCTTGATGATAATGGACTGAACCTC-3'(-2389 from TSS)

f

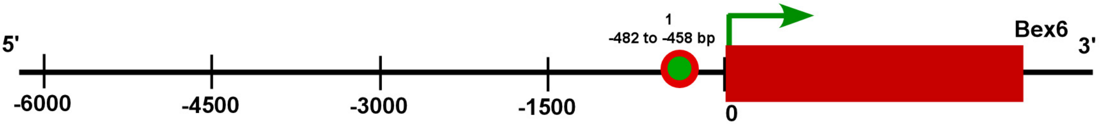

(-538 bp from TSS) 5'-
GACCTGGAAACACCTAGAAGTTCAATGTTCTCTTAAATTGAATGAGAATATAGGCAGGACTTGCATAATCAATGCATGCCC
AGAATATAAGCAGAGATATCATTAGACAGACAGTGC-3'(-421 from TSS)

Supplementary Fig. S5. Analysis of *Bex* genes promoter to locate possible p53 DNA binding elements as reported by El Deiry et al., 1992 and Veprintsev et al., 2008.

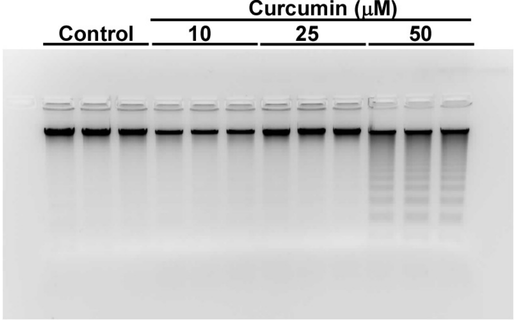

Supplementary Fig. S6. Original full-length gel image of Fig. 1e of manuscript file exhibiting curcumin induces DNA fragmentation in N2a cells at 24 hours of treatment. Equal amount of RNA free DNA from each sample were electrophoresed in 1% agarose gel and images were captured using XRS+ imager (Bio-Rad) and Image Lab software (version 3.0). Images were inverted and exported for publication at $\geq$ 600 DPI in tiff format. Image displayed in this figure is not modified by any image processing softwares after image acquisition.

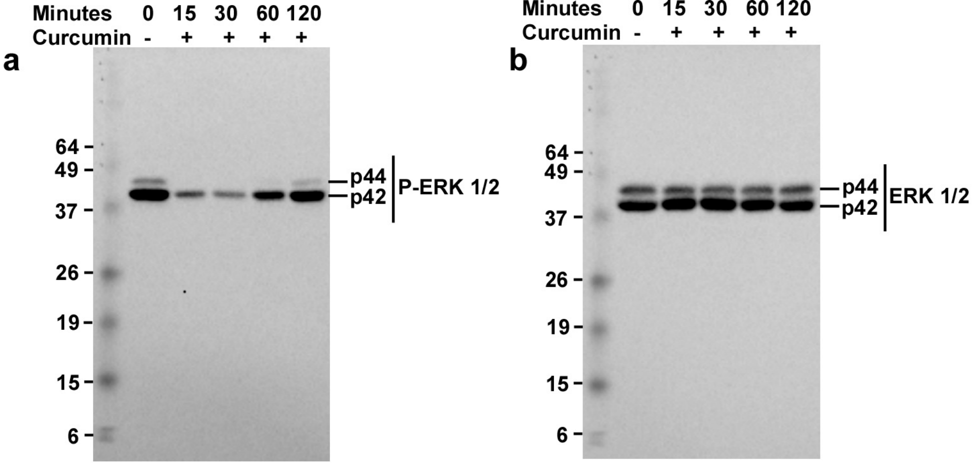

Supplementary Fig. S7. Original full-length images of immunoblots displayed in Fig. 2a of manuscript file. (**a**) Curcumin inactivated ERK1/2 in N2a cells. Blot was probed with anti-phospho-ERK 1/2 antibody and imaged using ChemiDoc XRS+ imager (Bio-Rad) and Image Lab software (version 3.0). (**b**) After imaging the blot was stripped and immunoblotted again with anti-ERK 1/2 antibody. Images displayed in this figure are not processed further by any image processing software.

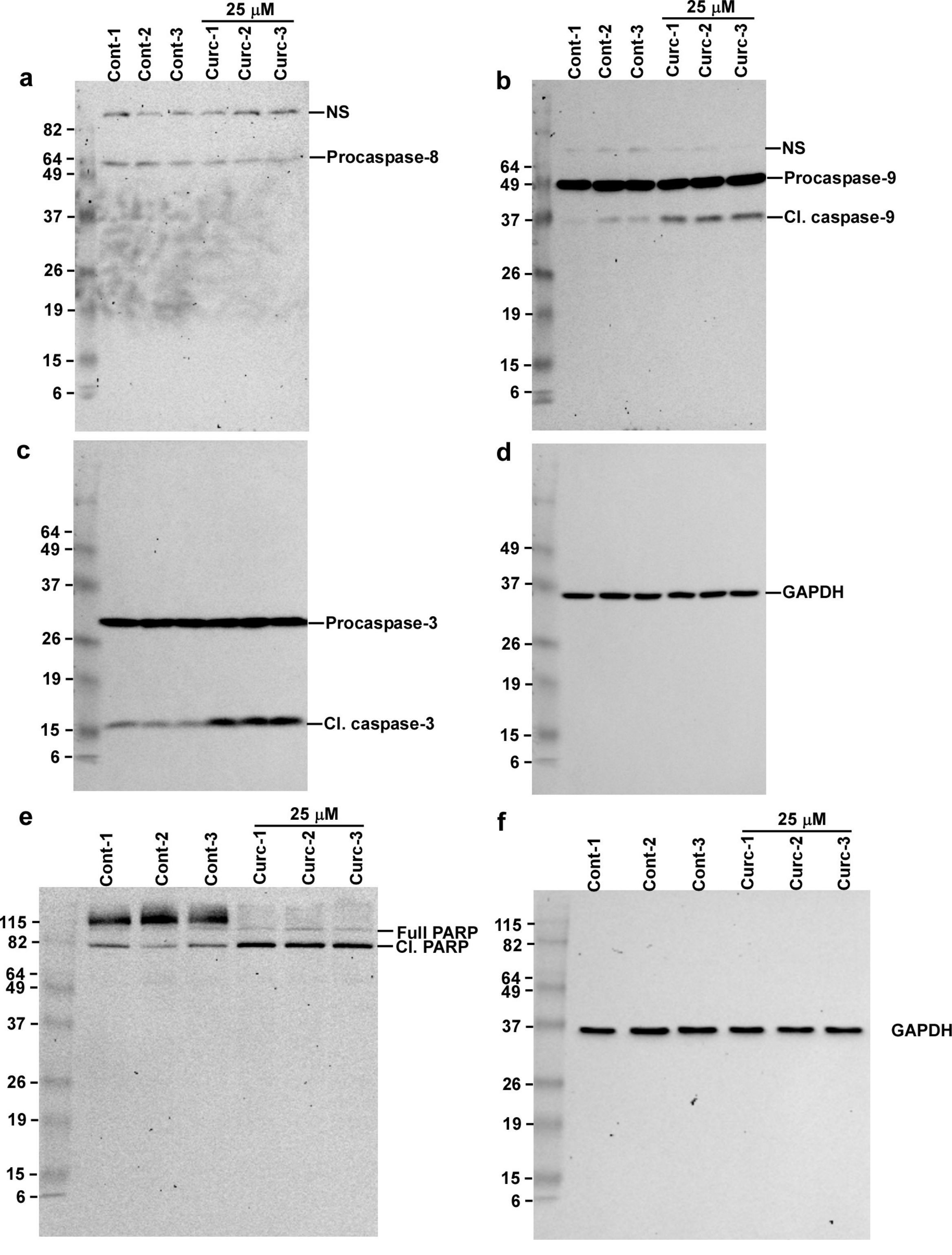

Supplementary Fig. S8. Original full-length images of caspase-8 blot (**a**), caspase-9 (**b**), caspase-3 (**c**) and respective GAPDH blot (**d**) as displayed in Fig. 2c. Full image of PARP-1 blot (**e**) and respective GAPDH blot (**f**) as displayed in Fig. 2f.

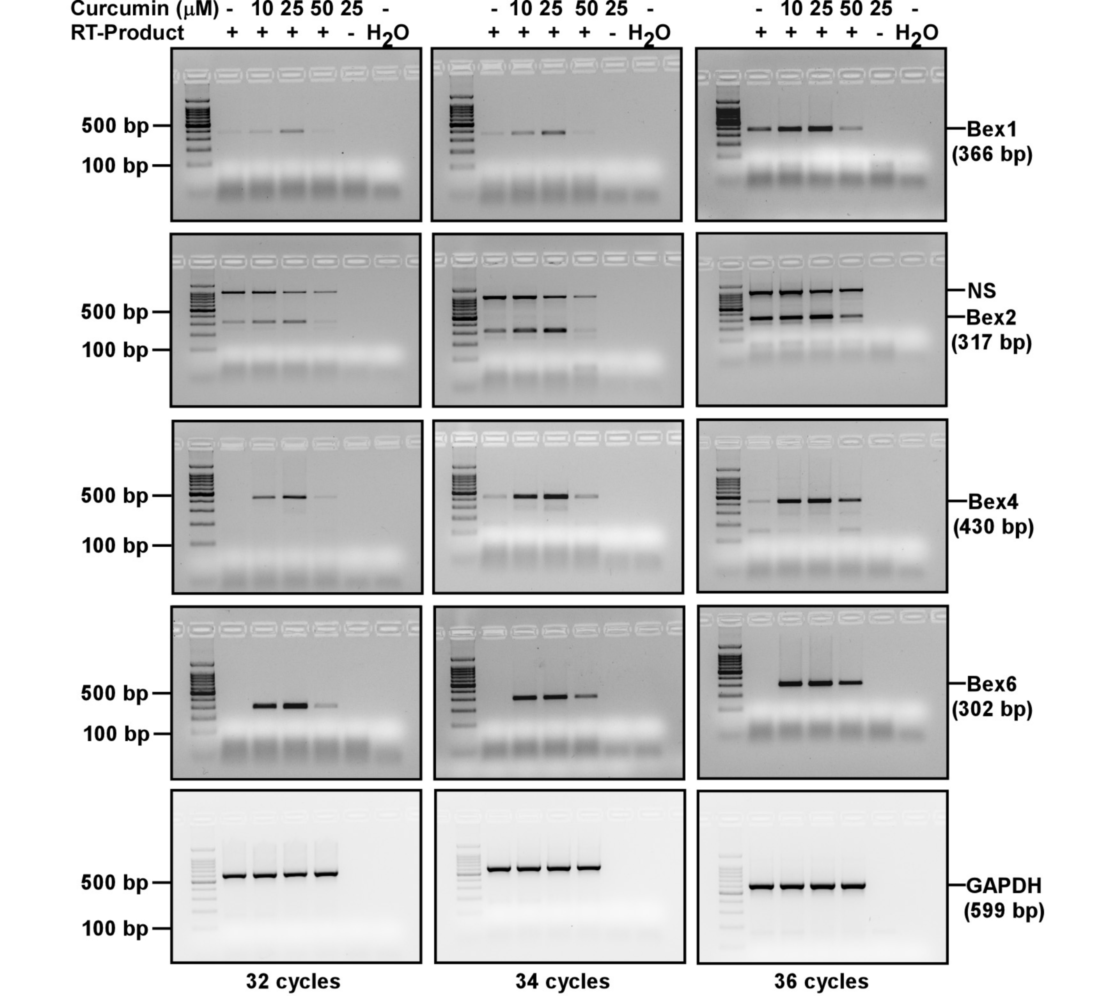

Supplementary Fig. S9. Original full-length images used for Fig. 3 of manuscript file, which show dose-dependent induction of *Bex* genes in curcumin treated N2a cells. Images displayed in this figure were not modified by any image processing software after image acquisition. Images displayed in Fig. 3 were cropped from these images but processed and displayed with identical settings.

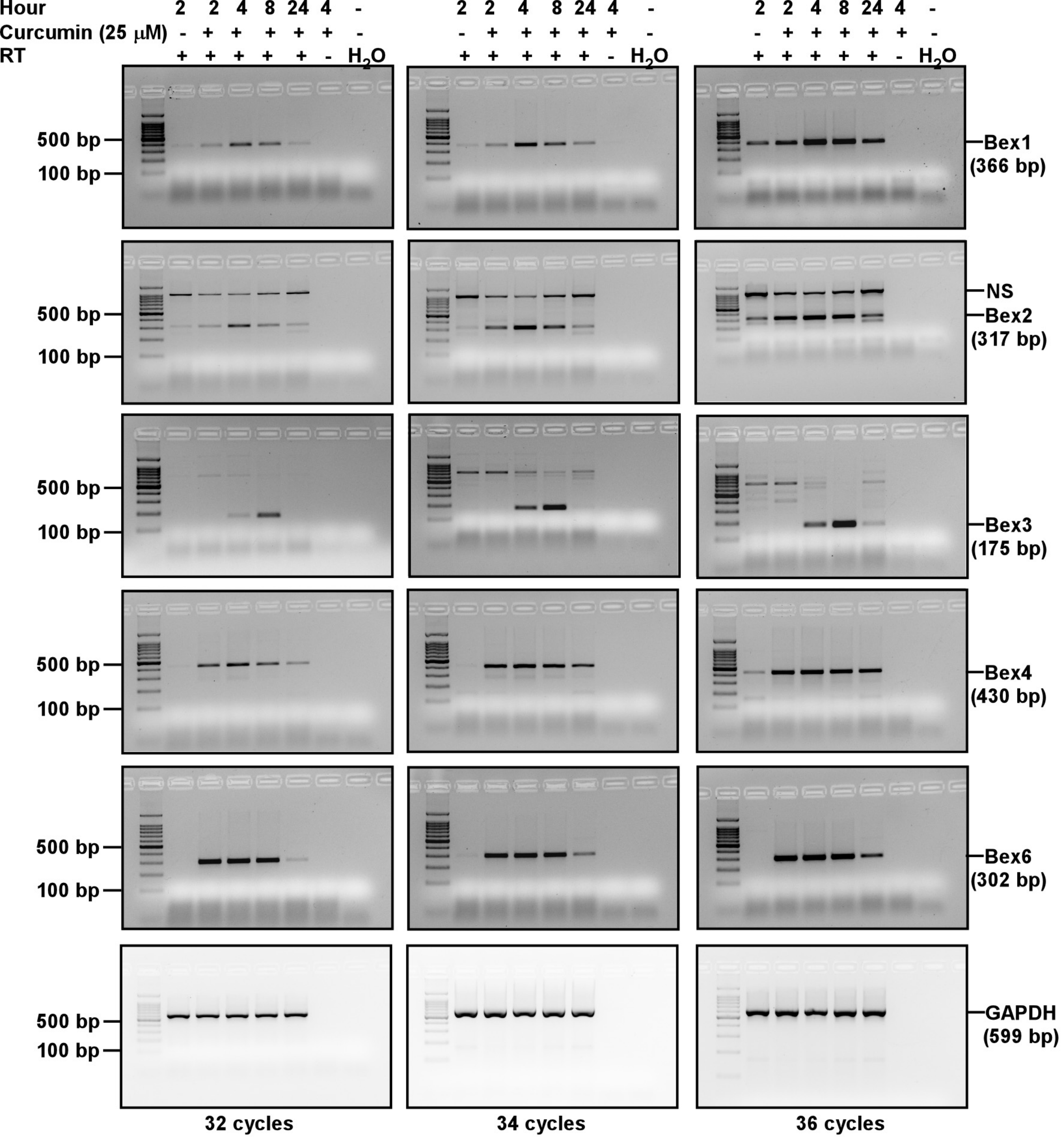

Supplementary Fig. S10. Original full-length images of gels used in Fig. 4 without any image processing after image acquisition show, a time-dependent activation of *Bex* genes in curcumin treated N2a cells. Images displayed in Fig. 4 were cropped from these images but processed and displayed with identical settings.

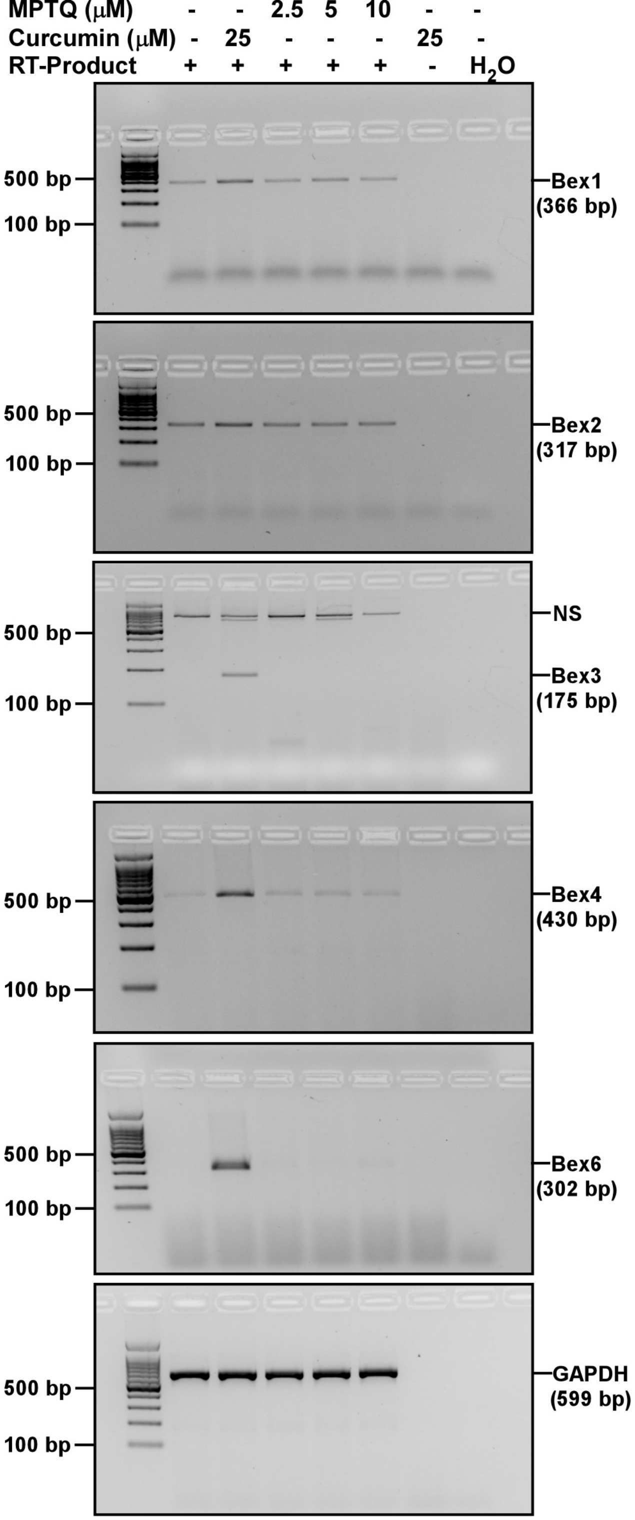

Supplementary Fig. **S11.** Original full-length agarose gel images used for Fig. 5 of manuscript file without any image processing after image acquisition show, curcumin as a specific inducer of all the *Bex* genes. Images displayed in Fig. 5 were cropped from these images but processed and displayed equally. NS: Non-specific.

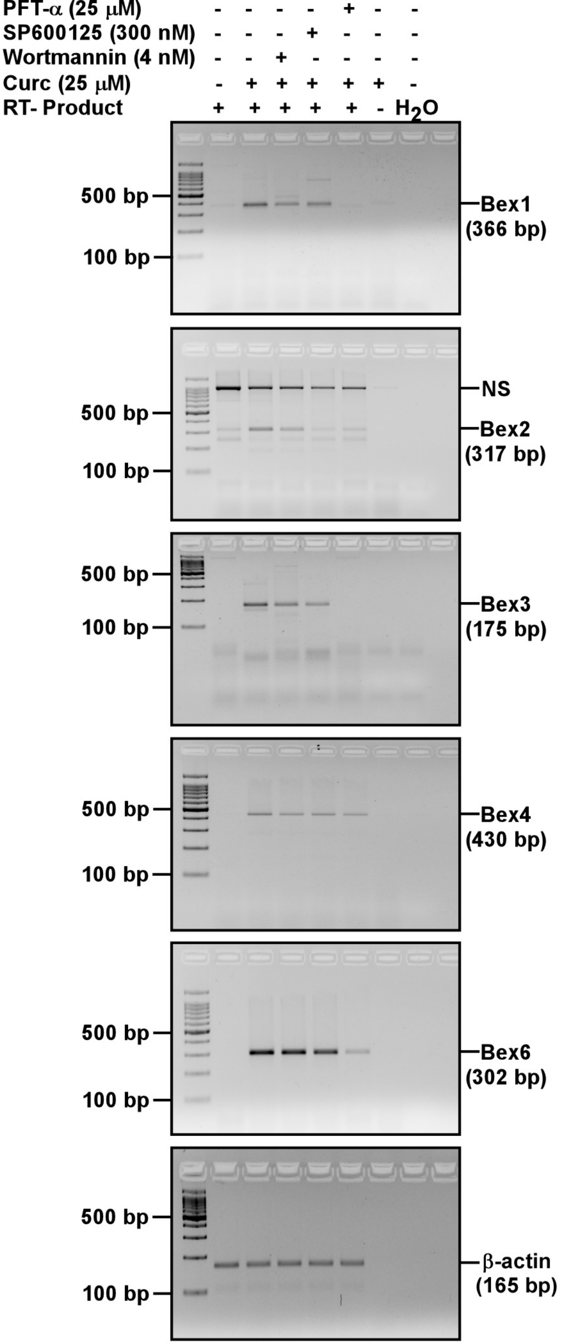

Supplementary Fig. S12. Original full-length gel images used for Fig. 6a of manuscript file show, cell signaling pathways regulating curcumin-mediated induction of *Bex* genes. Images of Bex1 RT-PCR products were captured at 200 milliseconds and Bex2, 3, 4, 6, and β-actin at 150 milliseconds using ChemiDoc XRS+ imager (Bio-Rad) and Image Lab software (version 3.0). Images were inverted and exported for publication at $\geq$ 600 DPI in tiff format. Images displayed in Fig. 6a were cropped from these images but processed and displayed with identical settings. NS: Non-specific.

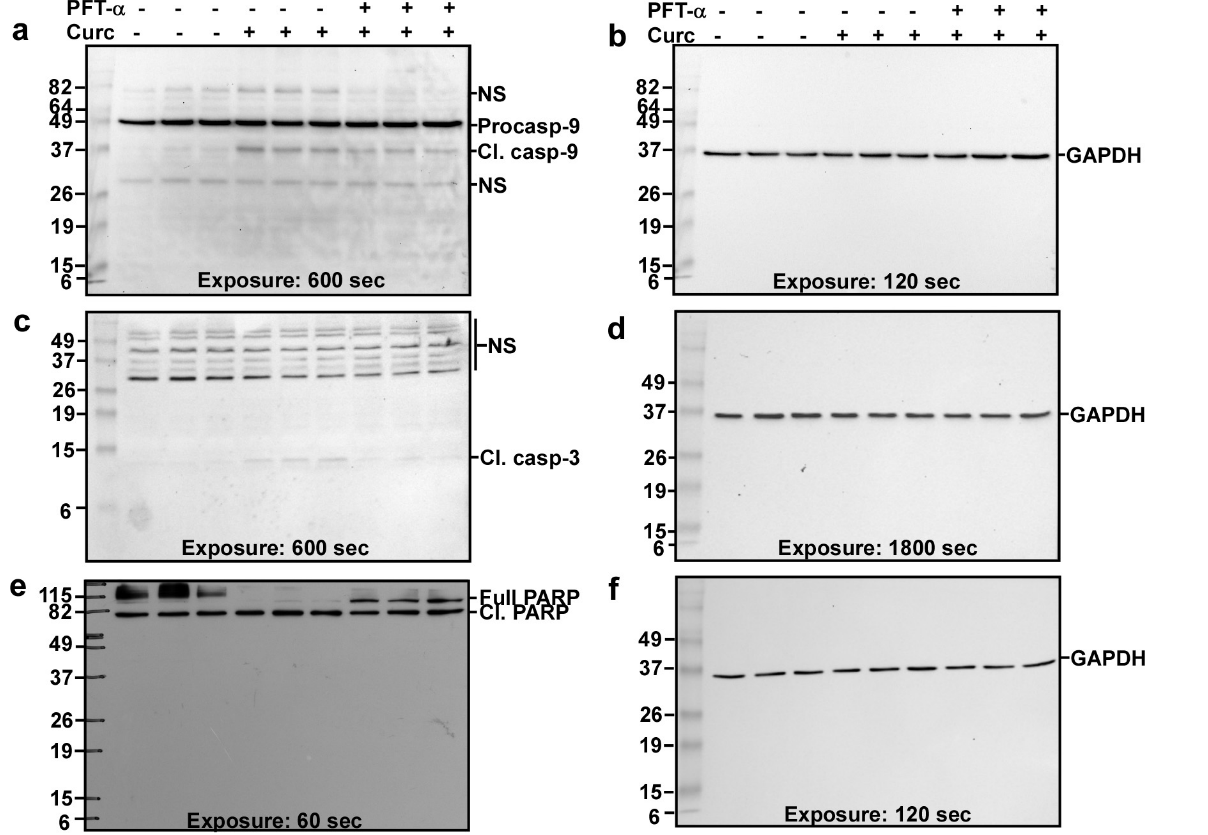

Supplementary Fig. S13. Original full-length gel images used for Fig. 6e of main manuscript file. Association of Bex genes induction with curcumin-mediated apoptotic N2a cells death. (**a**) Full image of blot hybridized with anti-caspase-9 antibody which detects both procaspase and cleaved caspase-9 protein and (**b**) respective GAPDH blot. (**c**) Full image of blot hybridized with anti-caspase-3 antibody which detects only cleaved caspase-3 protein. (**d**) GAPDH blot from samples derived from same experiment, processed in parallel. (**e**) Original image of blot hybridized with anti-PARP-1 antibody which detects both full and cleaved PARP-1 protein and (**f**) corresponding GAPDH blot. Images displayed in this figure are not modified by any image processing software after image acquisition and file export. NS: Non-specific.

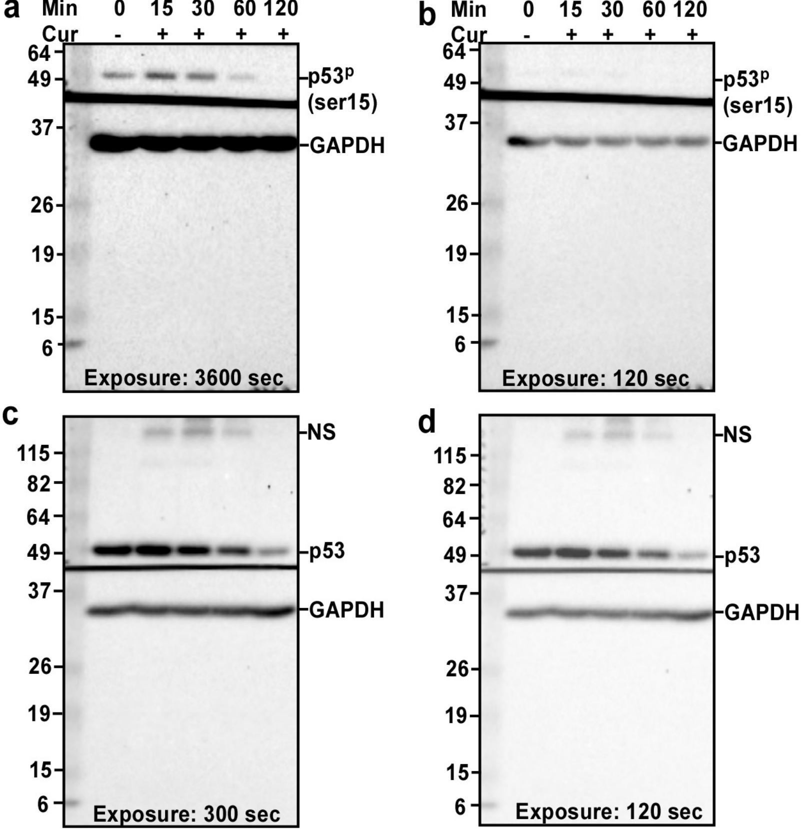

Supplementary Fig. S14. Original immunoblot images used in Fig. 7a of manuscript file demonstrating hyperphosphorylation of p53 at ser15 prior to *Bex* genes induction in curcumin treated N2a cells. Blot with 49 kDa was immunoblotted with either anti-phospho p53-ser15 or p53 antibody. Corresponding blots with 37 kDa were immunoblotted with GAPDH antibody. Images displayed in this figure are not modified by any image processing software after image acquisition and file export. NS: Non-specific.

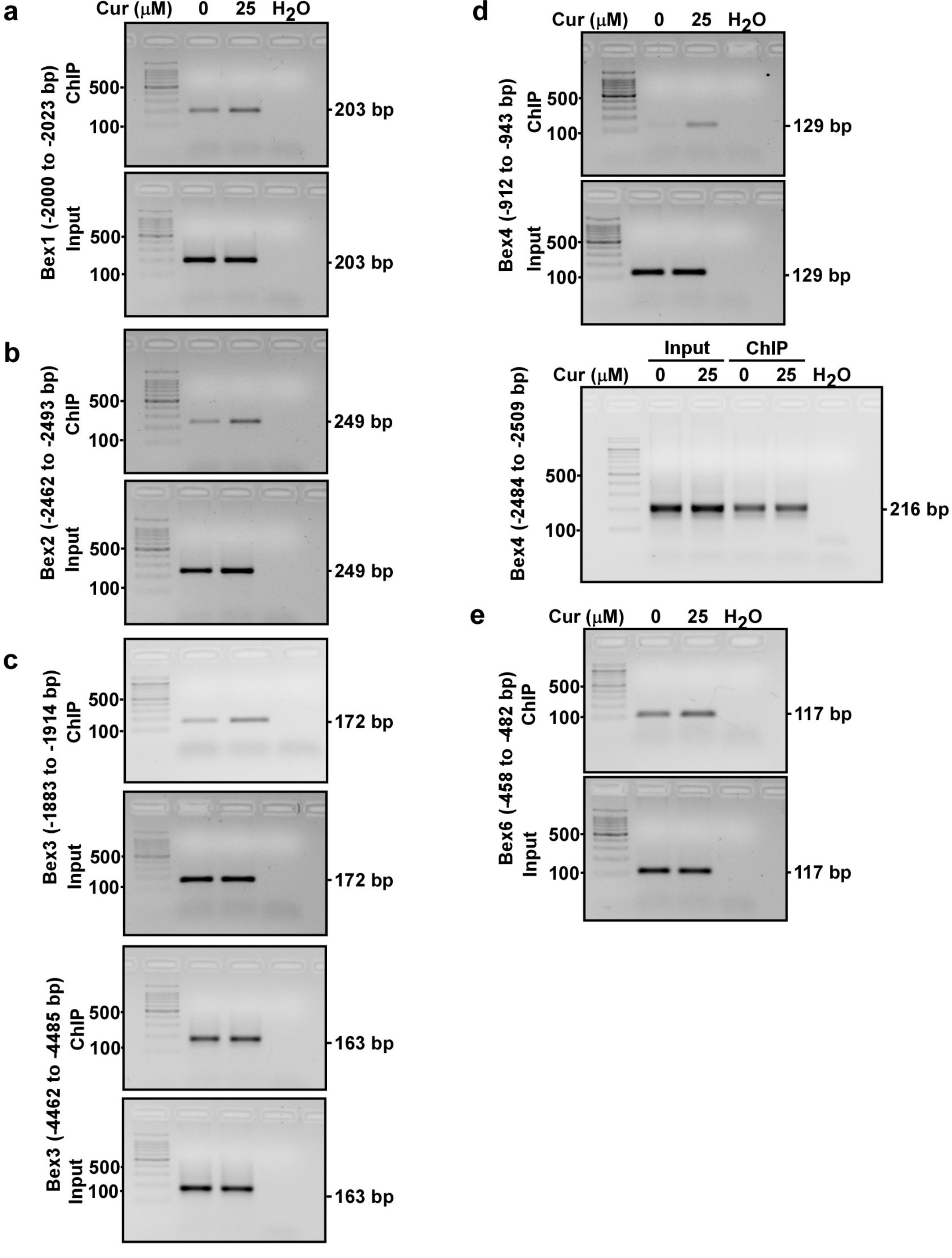

Supplementary Fig. S15. Original full-length gel images used in Fig. 7f-j clearly exhibit interaction of phospho-p53-ser15 onto the promoters of *Bex* genes by chromatin immunoprecipitation assay. (**a**), (**b**), (**c**), (**d**) and (**e**) Full-length gel images of Fig. 7f, 7g, 7h, 7i and 7j respectively. Images displayed in this figure are not modified by any image processing software after image acquisition and file export.

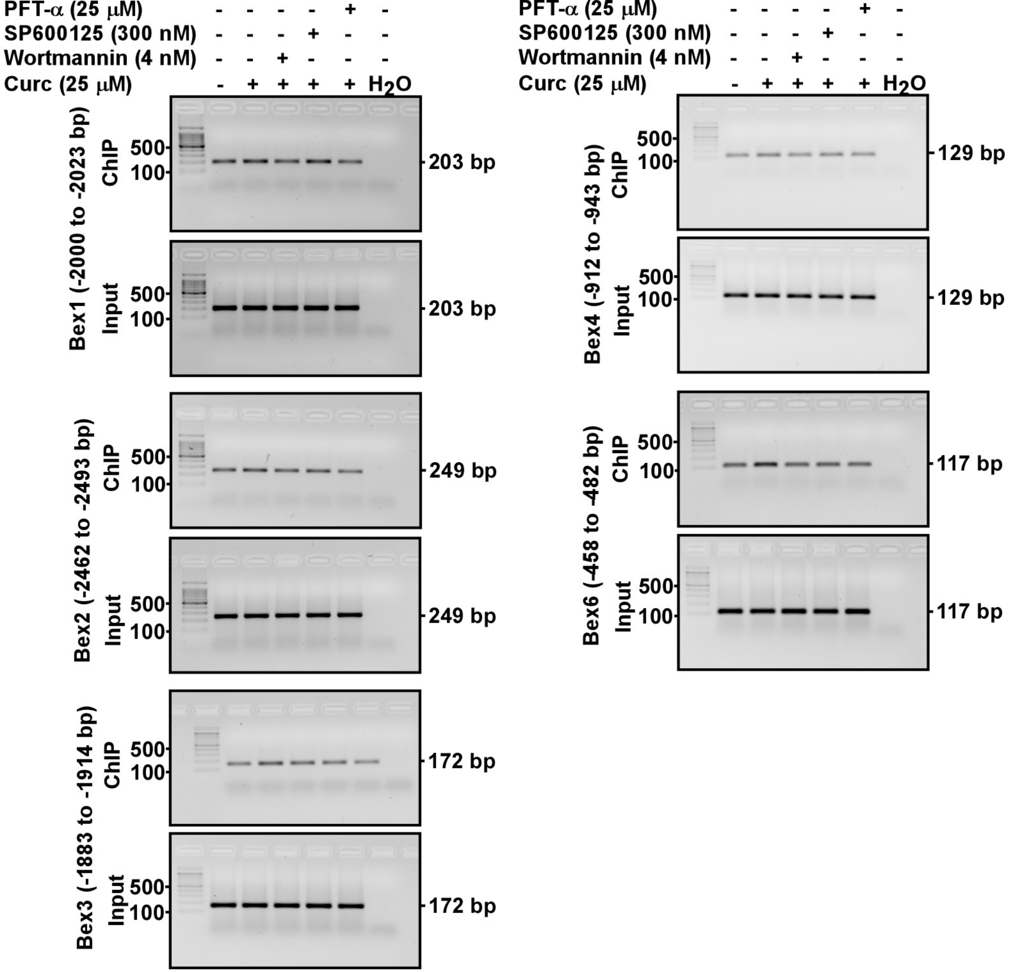

Supplementary Fig. S16. Original full-length gel images used in Fig. 7m demonstrate the effect of wortmannin (4 nM), SP600125 (300 nM) and pifithrin-α (25 μM) on phospho-p53-ser15 DNA binding activity to *Bex* genes at 30 minutes of curcumin (25 μM) treated N2a cells. PCR products of ChIP and input DNA were electrophoresed in 2% agarose gel, and images were captured at 200 milliseconds using ChemiDoc XRS+ imager (Bio-Rad) and Image Lab software (version 3.0), and exported for publication in tiff format. Images displayed in this figure are not modified by any image processing software after image acquisition and file export.

Supplementary Text

Legends to supplementary figures

Supplementary Fig. S1. Curcumin specifically induces all the *Bex* genes in N2a cells. (**a**) N2a cells were cultured, serum starved for 2 hours and treated with indicated concentrations of either MPTQ (10, 25 µM) or curcumin (25 µM). DMSO treated N2a cells were used as controls. After 4 hours of treatment, total RNA was isolated and expression of all the Bex mRNAs was studied by RT-PCR analysis. GAPDH was used as loading control. Densitometric analysis of Bex1 (**b**), Bex2 (**c**), Bex3 (**d**), Bex4 (**e**) and Bex6 (**f**) PCR products was performed and normalized with corresponding GAPDH band intensity. Values are displayed as histograms of mean±standard deviation from three independent experiments. p values displayed were calculated by using two-tailed, unpaired Student's t-test and *=p≤0.05 is considered statistically significant.

Supplementary Fig. S2. Curcumin-mediated induction of *Bex* genes is not associated with endoplasmic reticulum (ER) stress involved in N2a cells. (**a**) Approximately 70% confluent N2a cells were serum starved for 2 hours and then treated with either ER stress inducer tunicamycin (0.1 µg/ml) or curcumin (25 µM). Control cells were treated with equivalent amount of DMSO. Expression of all the Bex mRNAs was studied after 4 hours of treatment by RT-PCR analysis. GAPDH was used as loading control. Densitometric analysis of Bex1 (**b**), Bex2 (**c**), Bex3 (**d**), Bex4 (**e**) and Bex6 (**f**) PCR products was performed and normalized with corresponding GAPDH band intensity. Values are displayed as histograms of mean±standard deviation from three independent experiments. p values displayed were calculated by using two-tailed, unpaired Student's t-test and *=p≤0.05 is considered statistically significant.

Supplementary Fig. S3. Screening of cell signaling pathways involved in curcumin-mediated induction of *Bex* genes utilizing pharmacological inhibitors in N2a neuroblastoma cells. N2a cells were cultured, serum starved and treated with indicated concentrations of either PD98059 (MEK1/2

inhibitor), SB202190 (p38 MAP kinase inhibitor), SP600125 (JNK inhibitor), U0126 (MEK1/2 inhibitor), wortmannin (PI3 Kinase inhibitor) or pifithrin-α (p53 inhibitor) 30 minutes prior to curcumin (25 µM) treatment. Control cells were treated with equivalent amount of DMSO. After 4 hours of treatment, total RNA was isolated, DNase I treated and expression of Bex mRNAs was studied by RT-PCR analysis. GAPDH was used as loading control. (**a**) Pharmacological inhibitors were used at IC50 dose for initial screening of molecular mechanisms involved in curcumin-mediated Bex genes induction. (**b**) Densitometric analysis of Bex1, Bex2, Bex4 and Bex6 PCR products was performed, normalized with corresponding GAPDH band intensity and fold change was plotted as histograms. (**c**) Dose titration of selected inhibitors (wortmannin, SP600125 and pifithrin-α) against curcumin-mediated Bex gene induction. Densitometric analysis of Bex1 (**d**), Bex2 (**e**), Bex4 (**f**) and Bex6 (**g**) PCR products was performed and histograms were plotted as described above. Values are displayed as histograms of mean±standard deviation from three independent experiments. p values displayed were calculated by using two-tailed, unpaired Student's t-test and $* = p < 0.05$ is considered statistically significant.

Supplementary Fig. S4. Association of *Bex* genes with curcumin-mediated N2a cell apoptosis. (a) N2a cells were transfected with 200 pmol of Bex1, 2, 4 or 6 siRNA duplexes either individually or in combination for 72 hours followed by 25 µM of curcumin treatment for 24 hours. Scrambled siRNA transfected N2a were used as experimental control. Untransfected DMSO treated and only curcumin treated cells were used as negative and positive controls. (**a**) Fluorescent images from LIVE/DEAD assay were captured at center of the well using 4X objective lens and displayed with identical settings. (**b**) Integrated intensity of ethidium homodimer for each group was measured using metamorph software (version 7.7.0.0) and percent change in intensity is plotted as histograms which indicate Bex6 and Bex1 have maximal inhibitory effect followed by all siRNA, Bex4 and Bex2 in curcumin-mediated N2a cell death. Scrambled siRNA has no inhibitory effect.

Supplementary Fig. S5. Analysis of *Bex* genes promoter to locate possible p53 DNA binding elements. (**a**) DNA binding elements for p53 as reported by El Deiry et al., 1992 and Veprintsev et al., 2008 were used to locate potential p53 DNA binding elements in the promoter region of *Bex1* (**b**), *Bex2* (**c**), *Bex3* (**d**), *Bex4* (**e**) and *Bex6* (**f**) genes. Red colored sequence represents p53 binding site and green colored sequence represent flanking primers of the respective p53 binding site.

Supplementary Fig. S6. Original full-length gel image of Fig. 1e of manuscript file exhibiting curcumin induces DNA fragmentation in N2a cells at 24 hours of treatment. Equal amount of RNA free DNA from each sample were electrophoresed in 1% agarose gel and images were captured using ChemiDoc XRS+ imager (Bio-Rad) and Image Lab software (version 3.0). Images were inverted and exported for publication at ≥ 600 DPI in tiff format. Image displayed in this figure is not modified by any image processing softwares after image acquisition.

Supplementary Fig. S7. Original full-length images of immunoblots displayed in Fig. 2a of manuscript file. (**a**) Curcumin inactivated ERK1/2 in N2a cells. Blot was probed with anti-phospho-ERK 1/2 antibody and imaged using ChemiDoc XRS+ imager (Bio-Rad) and Image Lab software (version 3.0). (**b**) After imaging the blot was stripped and immunoblotted again with anti-ERK 1/2 antibody. Images displayed in this figure are not processed further by any image processing software.

Supplementary Fig. S8. Original full-length images of caspase-8 blot (**a**), caspase-9 (**b**), caspase-3 (**c**) and respective GAPDH blot (**d**) blots used in Fig. 2c. Original image of PARP-1 blot (**e**) and respective GAPDH blot (**f**) used in Fig. 2f. Images displayed in this figure are not processed further by any image processing software.

Supplementary Fig. S9. Original full-length images used for Fig. 3 of manuscript file, which show dose-dependent induction of *Bex* genes in curcumin treated N2a cells. Total RNA was isolated from N2a cells treated either with 10, 25 or 50 µM of curcumin or with equal amount of DMSO as controls at 2 hours. Five microgram of DNA-free RNA was reverse transcribed and Bex cDNAs were amplified either for 32, 34 or 36 PCR cycles. PCR products were resolved in 2% agarose gels and images for Bex1, Bex2, Bex4 and Bex6 were captured at 100 milliseconds of exposure and GAPDH at 50 milliseconds of exposure using ChemiDoc XRS+ imager (Bio-Rad) and Image Lab software (version 3.0). Images were inverted and exported for publication at ≥ 600 DPI in tiff format. Images displayed in this figure were not modified by any image processing software after image export. These original images show a single band of Bex1 amplicon (366 bp), Bex4 (430 bp), Bex6 (302 bp) and GAPDH (599 bp) as expected but two distinct bands for Bex2 (>1000 bp as non-specific amplicon and ~317 bp as specific amplicon). Amplicons of correct size were used for densitometric analysis. Images displayed in Fig. 3 were cropped from these images but processed and displayed with identical settings.

Supplementary Fig. S10. Original images of gels used in Fig. 4 without any image processing after image acquisition show, a time-dependent activation of *Bex* genes in curcumin treated N2a cells. Total RNA was isolated from N2a cells treated with 25 µM of curcumin at indicated time intervals. RT-PCR analysis of Bex cDNAs was performed at indicated PCR cycles. PCR products were resolved in 2% agarose gels and images for Bex1, Bex2, Bex4 and Bex6 were captured at 100 milliseconds of exposure and GAPDH at 50 milliseconds of exposure using ChemiDoc XRS+ imager (Bio-Rad) and Image Lab software (version 3.0). Images were inverted and exported for publication at ≥ 600 DPI in tiff format. These original images show a single band of Bex1 amplicon (366 bp), Bex4 (430 bp), Bex6 (302 bp) and GAPDH (599 bp) but two distinct amplicons for Bex2 (>1000 bp as non-specific amplicon and ~317 bp as specific amplicon) and a dominant ~175 bp Bex3 amplicon. Amplicons of correct size were used for

densitometric analysis. Images displayed in Fig. 4 were cropped from these images but processed and displayed with identical settings.

Supplementary Fig. S11. Original full-length agarose gel images used for Fig. 5a of manuscript file without any image processing after image acquisition show, curcumin as a specific inducer of all *Bex* genes. Bex1, 2, 3, 4, 6 and GAPDH cDNAs were amplified for 30, 30, 38, 35, 35 and 30 cycles respectively and resolved in appropriate agarose gel. Images of Bex1, 2 and 3 PCR products were captured at 200 milliseconds, Bex4 and 6 at 150 milliseconds, and GAPDH at 100 milliseconds using ChemiDoc XRS+ imager (Bio-Rad) and Image Lab software (version 3.0). Images were inverted and exported for publication at ≥ 600 DPI in tiff format. These original images show a single band of Bex1 (366 bp), Bex2 (317 bp), Bex4 (430 bp), Bex6 (302 bp) and GAPDH (599 bp) amplicon but distinctly two Bex3 amplicons (>1000 bp as non-specific amplification and ~175 bp as specific amplicon). Amplicons of correct size were used for densitometric analysis. Images displayed in Fig. 5a were cropped from these images but processed and displayed with similar settings. NS: Non-specific.

Supplementary Fig. S12. Original full-length gel images used for Fig. 6a of main manuscript file show, cell signaling pathways regulating curcumin-mediated induction of *Bex* genes. Approximately 80% confluent N2a cells were treated with wortmannin (4 nM), SP600125 (300 nM) or pifithrin-α (25 μM) for 30 minutes after two hours of serum starvation. Cells were then treated with either curcumin (25 μM) alone or along with these inhibitors. DMSO treated cells were used as controls. Total RNA was isolated at 4 hours of curcumin treatment. DNA-free RNA (5 μg) was used for RT-PCR analysis as described earlier, which demonstrates wortmannin, SP600125 and pifithrin-α inhibited curcumin-mediated induction of *Bex* genes. Images of Bex1 PCR products were captured at 200 milliseconds and Bex2, 3, 4, 6, and β-actin at 150 milliseconds using ChemiDoc XRS+ imager (Bio-Rad) and Image Lab software (version 3.0).

Images were inverted and exported for publication at ≥ 600 DPI in tiff format. Amplicons of correct size were used for densitometric analysis. Images displayed in Fig. 6a were cropped from these images but processed and displayed with identical settings. NS: Non-specific.

Supplementary Fig. S13. Original full-length gel images used for Fig. 6e of main manuscript file demonstrating association of *Bex* genes induction with curcumin-mediated N2a cells apoptosis. Cell lysates were prepared after 24 hours either from DMSO or curcumin (25 μM) alone or along with pifithrin-α (25 μM) treated N2a cells. Western blotting was performed using antibodies against caspase-9, caspase-3 or PARP1. GAPDH immunoblotting was used as loading and transfer control. Time-lapse chemiluminescent images of immunoblots were captured using ChemiDoc XRS+ imager (Bio-Rad) and Image Lab software (version 3.0), merged with bright field image of blot with molecular weight marker, and exported for publication in tiff format. Blot hybridized with anti-PARP-1 antibody was developed using X-ray film. (**a**) Original image of caspase-9 immunoblot, which detects both procaspase-9 and cleaved caspase-9. (**b**) Blot was stripped and immunoblotted with GAPDH antibody. (**c**) Full image of blot hybridized with anti-caspase-3 antibody, which detects only cleaved caspase-3 protein. (**d**) GAPDH blot from samples derived from same experiment and processed in parallel. (**e**) Full image of blot hybridized with anti-PARP-1 antibody which detects both full and cleaved PARP-1 protein and (**f**) respective GAPDH blot. Images displayed in this figure are not modified by any image processing software after image acquisition and export. Images displayed in Fig. 6e were cropped from these images but processed and displayed with identical settings. NS: Non-specific.

Supplementary Fig. S14. Original immunoblot images used in Fig. 7a of main manuscript file demonstrating hyperphosphorylation of p53 at ser15 prior to *Bex* genes induction in curcumin treated N2a cells. Each blot was cut between 49 and 37 kDa molecular weight marker (pre-stained protein marker,

Invitrogen). Blot with 49 kDa was immunoblotted with either anti-phospho p53-ser15 or p53 antibody. Corresponding blots with 37 kDa were immunoblotted with GAPDH antibody. Time lapse chemiluminescent images of immunoblots were captured using ChemiDoc XRS+ imager (Bio-Rad) and Image Lab software (version 3.0), merged with bright field image of blot with molecular weight marker and exported for publication in tiff format. Higher exposure (**a**) and lower exposure (**b**) of phospho-p53-ser15 and respective GAPDH blot. Higher (**c**) and lower exposure (**d**) of p53 and respective GAPDH blots. Images displayed in this figure are not modified by any image processing software after image acquisition and file export. Images displayed in Fig. 7a were cropped from these images but processed and displayed with identical settings. NS: Non-specific.

Supplementary Fig. S15. Original full-length gel images used in Fig. 7f-j clearly exhibit interaction of phospho-p53-ser15 onto the promoters of *Bex* genes by chromatin immunoprecipitation (ChIP) assay. DNA from both ChIP and original lysate (Input) were used to amplify region containing p53 binding element found in the promoter region (-1 to -6000 bp upstream to transcription start site) of *Bex* genes using specific primers. Amplified DNA were electrophoresed in a 2% agarose gel and imaged at 200 milliseconds except Bex4 (-2484 to -2509 bp) which was captured at 100 milliseconds using ChemiDoc XRS+ imager (Bio-Rad) and Image Lab software (version 3.0) and exported for publication in tiff format. (**a**), (**b**), (**c**), (**d**) and (**e**) Full-length gel images of Fig. 7f, 7g, 7h, 7i and 7j respectively. Images displayed in this figure are not modified by any image processing software after image acquisition and file export. Images displayed in Fig. 7f-j were cropped from these images but processed and displayed with identical settings.

Supplementary Fig. S16. Original full-length gel images used in Fig. 7m demonstrate the effect of wortmannin (4 nM), SP600125 (300 nM) and pifithrin-α(25 μM) on phospho-p53-ser15 DNA binding

activity to *Bex* genes at 30 minutes of curcumin (25 µM) treated N2a cells. PCR products of ChIP and input DNA were electrophoresed in 2% agarose gel, and images were captured at 200 milliseconds using ChemiDoc XRS+ imager (Bio-Rad) and Image Lab software (version 3.0), and exported for publication in tiff format. Images displayed in this figure are not modified by any image processing software after image acquisition and file export. Images displayed in Fig. 7m were cropped from these images but processed and displayed with identical settings.

Equipment and settings.

All agarose gel images were captured at defined exposure time using nucleic acid gel application for ethidium bromide in Image lab software (version 3.0) supplied with ChemiDoc XRS_+ gel doc system (Bio-Rad). The molecular weight marker, all controls and treated samples were imaged in the same gel. The images were displayed in grey scale with similar settings for a specific gene, inverted and exported for publication as a tiff file.

For western blot imaging, the blots were either cut and hybridized with multiple antibodies or processed as full-length blot with single antibody immunoblotting. Blots containing prestained molecular weight marker, controls and treated samples were imaged as a single unit using application for colorimetric imaging in Image lab software (version 3.0) supplied with ChemiDoc XRS_+ gel doc system (Bio-Rad). Time-lapse chemiluminescence images were captured using same system. Alternatively, few blots were also developed using X-ray films and scanned using ChemiDoc XRS_+ gel doc system. The bright field image of blot was merged with chemiluminiscent image, scaled to same settings, and exported to tiff format file. All the tiff images were cropped using canvas software for figure preparation. Brightness and contrast settings were applied equally across entire image using canvas software. Images displayed in supplementary figures containing full length blot/gel images were not modified by any means further.

Fluorescent images were captured using 40X or 20X or 4X plan fluor lens in Nikon Ti eclipse microscope supported by Metamorph software (version 7.7.0.0) at same exposure settings across all samples of one experiment and preferably in one sitting. Fluorochromes such as Alexa 594, 647 or DAPI and respective filters were used to capture images using monochromatic camera. All the images were calibrated as per magnification, displayed at same scale settings, color combined using Metamorph software and saved as tiff files. The tiff images were cropped and processed equally using canvas software for figure preparation.
